# Genus *Stachys*: A Review of Traditional Uses, Phytochemistry and Bioactivity

**DOI:** 10.3390/medicines7100063

**Published:** 2020-09-29

**Authors:** Ekaterina-Michaela Tomou, Christina Barda, Helen Skaltsa

**Affiliations:** Department of Pharmacognosy and Chemistry of Natural Products, Faculty of Pharmacy, School of Health Sciences, National & Kapodistrian University of Athens, Panepistimiopolis, Zografou, 15771 Athens, Greece; ktomou@pharm.uoa.gr (E.-M.T.); cbarda@pharm.uoa.gr (C.B.)

**Keywords:** *Stachys* L., traditional uses, pharmacological activities, phytochemicals, bioactive compounds

## Abstract

**Background**: The genus *Stachys* L. (Lamiaceae) includes about 300 species as annual or perennial herbs or small shrubs, spread in temperate regions of Mediterranean, Asia, America and southern Africa. Several species of this genus are extensively used in various traditional medicines. They are consumed as herbal preparations for the treatment of stress, skin inflammations, gastrointestinal disorders, asthma and genital tumors. Previous studies have investigated the chemical constituents and the biological activities of these species. Thus, the present review compiles literature data on ethnomedicine, phytochemistry, pharmacological activities, clinical studies and the toxicity of genus *Stachys*. **Methods**: Comprehensive research of previously published literature was performed for studies on the traditional uses, bioactive compounds and pharmacological properties of the genus *Stachys*, using databases with different key search words. **Results**: This survey documented 60 *Stachys* species and 10 subspecies for their phytochemical profiles, including 254 chemical compounds and reported 19 species and 4 subspecies for their pharmacological properties. Furthermore, 25 species and 6 subspecies were found for their traditional uses. **Conclusions**: The present review highlights that *Stachys* spp. consist an important source of bioactive phytochemicals and exemplifies the uncharted territory of this genus for new research studies.

## 1. Introduction

The genus *Stachys* L., a large member of the Lamiaceae family, comprises more than 300 species, dispersing in temperate and tropical regions of Mediterranean, Asia, America and southern Africa [1,2,3]. Up to now, the most established and comprehensive classification of the genus is introduced by Bhattacharjee (1980), categorizing into two subgenera *Betonica* L. and *Stachys* L. [2,3]. The subgenus *Stachys* includes 19 sections, while the subgenus *Betonica* comprises 2 sections [1]. However, the two subgenera present important botanical and phytochemical differences which differentiate them [1,4,5].

*Stachys* species grow as annual or perennial herbs or small shrubs with simple petiolate or sessile leaves. The number of verticillate ranges from four to many-flowered, usually forming a terminal spike-like inflorescence. Calyx tubes are tubular-campanulate, 5 or 10 veined, regular or weakly bilabiate with five subequal teeth. Corolla has a narrow tube, 2-lipped; upper lip flat or hooded and generally hairy, while the lower lip is 3-lopped and glabrous to hairy. The nutlets are oblong to ovoid, rounded at apex [6].

The genus name derived from the Greek word «stachys (=στάχυς) », referring to the type of the inflorescence which is characterized as “spike of corn” and resembles to the inflorescences of the species of genus *Triticum* L. (Gramineae). In ancient times, the name “stachys” referred mainly to the species *Stachys germanica* L. whose inflorescence is like an ear and is covered with off-white trichome [7]. The Latin name of the genus is trifarium (=tomentose) [8].

Historically, Dioscorides mentioned the species *S. germanica* L. with the name “stachys” [9]. However, in late Byzantine era, ‘Nikolaos Myrepsos’ included some species of the genus *Stachys* (*S. germanica* L., *S. officinalis* (L.) Travis, *S. alopecuros* (L.) Benth.) in his medical manuscript “Dynameron”. Precisely, *S. officinalis* and *S. alopecuros* were probably included in 11 recipes, under the names vetoniki, drosiovotanon, lauriole, kakambri, while *S. germanica* was added in 1 recipe referred as stachys [10].

Many species of the genus are extensively used in traditional medicine of several countries, having various names. For instance, the species *S. recta*, known as yellow woundwort, is called as “erba della paura” (=“herb that keeps away fear”) in Italy, attributing to the anxiolytic properties of its herbal tea, while *S. lavandulifolia* Vahl is called as “Chaaye Koohi” in Iran [11,12,13]. In addition, herbal preparations of *Stachys* spp. are widely consumed in folk medicine to treat a broad array of disorders and diseases, including stress, skin inflammations, stomach disorders and genital tumors [3,14,15]. Specially, the herbal teas of these plants, known as “mountain tea”, are used for skin and stomach disorders [12,16]. The latter common name could lead to a misinterpretation since the herbal remedies of any *Sideritis* species are globally known with the same name.

In the international literature, *Stachys* species have been broadly studied through several phytochemical and pharmacological investigations, justifying their ethnopharmacological uses. Of special pharmacological interest are considered the anti-inflammatory, antioxidant, analgesic, renoprotective, anxiolytic and antidepressant activity [3,17,18,19]. The range of the therapeutic properties attributed to these species have been associated to their phytochemical content. Therefore, genus *Stachys* has received much attention for the screening of its bioactive secondary metabolites from different plant parts. In general, more than 200 compounds have been isolated from this genus, belonging to the following important chemical groups; terpenes (e.g., triterpenes, diterpenes, iridoids), polyphenols (e.g., flavone derivatives, phenylethanoid glycosides, lignans), phenolic acids and essential oils [3,5,14,20,21,22].

Consequently, plants of genus *Stachys* are considered a great source of phytochemicals with therapeutic and economic applications. Given the increasing demand for natural products, many *Stachys* species have been cultivated for uses in traditional medicine, in food market, in cosmetic industry and for ornamental reasons [21,22]. Despite the widely uses of the specific species and the large amount of research studies, there has been no recent comprehensive review including all the latest data of the specific genus and its contribution in medicine. Up to now, the available reviews are centered to the phytochemical profile and biological activities of *Stachys* spp. in correlation to chemotaxonomy approach [3,21,22,23]. Thus, this review summarizes the current state of knowledge on the traditional uses, phytochemistry, pharmacological activities, clinical studies and toxicity of the genus *Stachys* L.

## 2. Materials and Methods

A comprehensive search on previous studies was conducted on scientific databases such as PubMed, Scopus, Google scholar and Reaxys, including the years 1969–2020. The search terms “Stachys”, “Stachys compounds”, “Stachys phytochemicals”, “Stachys pharmacological” and “Stachys traditional uses” were used for data collection. Searches were performed for other potential studies by manual screening references in the identified studies. In total, 161 publications describing the traditional uses, bioactive compounds, pharmacological properties and the toxicity of the genus *Stachys* were included, excluding articles focuses on taxonomy, botany and agronomy. The traditional medicinal uses of *Stachys* species were reported in Table 1, while the isolated specialized products were categorized by species in Table 2, Table 3, Table 4, Table 5, Table 6, Table 7, Table 8, Table 9, Table 10, Table 11, Table 12, Table 13, Table 14 and Table 15, with the attempt of the discrimination between publications describing metabolites′ isolation (including NMR data) or identification/screening (by means of HPLC, LC-MS, etc.). The chemical structures of the bioactive compounds were showed in Table 16, Table 17, Table 18, Table 19, Table 20, Table 21, Table 22, Table 23, Table 24, Table 25, Table 26, Table 27, Table 28 and Table 29. The reported biological activities of extracts/compounds of the last five years were mentioned by *Stachys* species in Table 30. The general characteristics of the analyzed studies in the current review are showed in Table 31. According to recent publications which support the division of the genus *Stachys* based on Bhattacharjee (1980), the classification in the present review is formed on this latter study. The species name and their synonyms are quoted as reporting in databases “Plant list” or “Euro + Med” or “IPNI” [24,25,26].

## 3. Traditional Medicinal Uses of Genus *Stachys*

Several *Stachys* spp. have been used in various ethnomedicines for thousands of years. A plethora studies mentioned their diverse traditional medicinal uses. In the current review, a detailed description of the available data of the traditional uses of *Stachys* spp. is shown in Table 1, reporting 25 species and 6 subspecies of this genus. A careful overview of the specific table reveals that the ethnomedicinal use of *Stachys* spp. is particularly in the area covering of Mediterannean to Iran. Most of the species are consumed as herbal teas for the treatment of infections, common cold, gastrointestinal disorders, inflammation, skin disorders/wounds, asthma and anxiety.

The species *S. affinis* is widely used in Chinese traditional medicine for several uses such as common cold, heart disease, pain relief, antioxidant activity, ischemic brain injury, dementia and gastrointestinal related diseases [27,28,29,30]. Another species applied in Chinese folk medicine is *S. geobombycis*, known as DongChongXiaCao, which is recommended as tonic and interestingly, this species is also used in Europe and Japan [22].

In Iran, several species are applied as traditional therapeutic agents in various conditions, including *S. acerosa* [31], *S. fruticulosa* [32], *S. byzantina* (known in Farsi as “lamb’s ear” or “lamb’s tongue” or “sonbolehe noghrehi” or “zabanehe bare”) [33,34,35], *S. inflata* (local names; ′′poulk′′ or “Ghol-e-Argavan”) [31,36,37], *S. lavandulifolia* (known as “Chaaye Koohi”) [12,13,31,38,39,40,41,42,43,44], *S. pilifera* [31,45], *S. schtschegleevii* [32,34,46], *S. sylvatica* [47] and *S. turcomanica* [34]. Of considerable interest, *S. sylvatica* (common name “hedge woundwort”) is recommended for the treatment of women with polycystic ovary syndrome (PCOS) [47].

Furthermore, in Turkish folk medicine, the species *S. cretica* subsp. *anatolica*, *S. cretica* subsp. *mersinaea*, *S. iberica* subsp. *georgica*, *S. iberica* subsp. *stenostachya, S. kurdica*, *S. lavandulifolia* and *S. obliqua* are used mainly to treat colds, cough, stomach ache and as antipyretic agents, while *S. sylvatica* is applied in cardiac disorders [22,48,49,50].

In Italy, the infusions of the leaves of *S. annua* and *S. recta* are used to wash the face to reveal headache [51], whereas the aerial parts of the subspecies *S. annua* subsp. *annua*, known as “stregona annual” or “erba strega”, are consumed as anti-catarrhal, febrifuge, tonic and vulnerary [52]. The decoction of the aerial parts of *S. recta* is also consumed as purative and for bad luck/spirit [53,54]. Interestingly, *S. annua* and *S. arvensis,* as well as the subspecies *S. recta* subsp. *recta* are applied against evil eye [11,51,52,55]. Moreover, in an area of central Italy, the species *S. officinalis* is used as oily extract to treat wounds and to dye wood yellow [29,54]. To be mentioned that *S. recta* is listed in the European Pharmacopeia, as well as *S. officnalis* is mentioned in Anthroposophic Pharmaceutical Codex (APC) [22]. However, Gören (2011) reported that some species (e.g., *S. annua, S. recta and S. sylvatica*) have been mentioned to be poisonous [22].

In North Greece, the infusion and decoction of *S. iva* are consumed against common cold and gastrointestinal disorders [56]. In addition, Fazio et al. (1994) reported different formulations of the Greek species *S. mucronata* applied in Greek tradition medicine. Precisely, the decoction of this species is consumed as an antirheumatic and antineuralgic agent, as well as the juice of fresh leaves is applied in wounds and ulcers. Moreover, the infusion of fresh leaves has antidiarrhoic effect, while the infusion of roots is purgative [57].

In addition to traditional medicinal uses, some species of genus *Stachys* are also consumed as edible plants, vegetables and food additives like the tubers of *S. affinis* (known as Chinese artichoke/chorogi; China/Japan) in China and Japan [22,27], the aerial parts of *S. lavandulifolia* in Iran [31], or the *S. palustris* in Poland [22,58]. The latter species is also included in the diet in Sweden, Ukraine and Great Britain [22]. Moreover, the dried powder of *S. palustris* is used as an additive for bread in Europe, thus it is known as “mayday flour” [22].

The infusion of the aerial parts of *Stachys* sp. LAM is used as traditional remedy for colic, gases and swollen stomach in Peru [22,59]. It is noteworthy to mention that a few species have been used in veterinary such as *S. germanica* and *S. officinalis* [30,54].

## 4. Chemical Composition

Various non-volatile chemical constituents have been reported from different species of genus *Stachys*, categorizing into important chemical groups including fatty acids, alkaloids (e.g., stachydrine, turiaine), triterpenes, phytosterols, phytoecdysteroids, diterpenes, iridoids, flavonoids, phenylpropanoid glucosides, acetophenones, phenylethanoid glycosides, lignans, phenolic acids, megastigmanes and polysaccharides [3,20,21,23,67]. The present survey was focused on all the above groups, excluded fatty acids and alkaloids due to the limited available studies. This section summarizes the phytochemicals from the genus *Stachys* which are mainly responsible for its pharmacological benefits, presented in Table 2, Table 3, Table 4, Table 5, Table 6, Table 7, Table 8, Table 9, Table 10, Table 11, Table 12, Table 13, Table 14 and Table 15. To be mentioned that large number of phytochemicals were mainly discovered from the aerial parts, leaves and a few were found in stems and roots.

### 4.1. Flavonoids

The genus *Stachys* consists a rich source of flavonoids. Accumulating studies have reported the several types of flavonoids occurring in *Stachys* spp., including flavones (Table 2 and Table 16), poly-methylated flavones (Table 3 and Table 17), flavonols (Table 4 and Table 18), flavanones (Table 5 and Table 19) and one biflavonoid (Table 6 and Table 20).

Regarding the flavone derivatives (Table 2 and Table 16), 18 flavone 7-O-acetylallosylglucosides were mentioned in the most species of subgenus *Stachys* (31 species). The flavone 7-O-glucosides were also found in many species through the two subgenera. Marin et al. (2004) reported that tricetin 3′,4′,5′-trimethyl-7-O-glucoside (**62**) consists a chemotaxonomic marker for the subgenus *Betonica* [5]. Precisely, selgin 7-O-glucoside (**59**), tricin 7-O-glucoside (**61**) and tricetin 3′,4′,5′-trimethyl-7-O-glucoside (**62**) were identified from the leaves of three species of the latter subgenus; *S. alopecuros* (section Betonica), *S. officinalis* (section Betonica) and *S. scardica* (section Macrostachya) [5]. Furthermore, derivatives of apigenin *p*-coumaroyl glucosides and chrysoeriol *p*-coumaroyl glucosides were reported in *Stachys* species, though some *p*-coumaroyl glucosides (not determined) were also identified [5,75]. To be mentioned that chrysoeriol 7-*O-*glucoside (**43**), chrysoeriol *p*-coumaroyl glucosides (**46,47**) and chrysoeriol 7-O-[6″′-O-acetyl-allosyl]-(1→2)-glucoside (stachyspinoside) (**44**) were mainly isolated from wild Greek taxa of the subgenus *Stachys* [3,77,98,99,102], apart from the Greek species *S. ionica* [20], *S. tetragona* [100] and the cultivated species *S. iva* [56]. Nazemiyeh et al. (2006) investigated the phytochemical profile of the stems of *S. schtschegleevii*, reporting four flavonoids, among them were also two *p*-coumaroyl derivatives of apigenin and chrysoeriol [74]. Moreover, flavone 7-O-mannosylglucosides were reported from the two species *S. atherocalyx* (section Eriostomum) and *S. spectabilis* (section Olisia) [72,89,90]. Few flavone C-glucosides were mentioned in the species *S. aegyptiaca* (subg. *Stachys*; sect. Ambleia), *S. officinalis* (subg. *Betonica*; sect. Betonica), and *S. scardica* (subg. *Betonica*; sect. Macrostachya) [5,68,104]. Zinchenko (1973) reported the existence of two derivatives of methoxybaicalein, namely palustrin (**63**) and palustrinoside (**64**), from the species *S. palustris* of subgenus *Stachys* (section Stachys) [104]. Notably, the subterranean organs of *S. annua* were investigated and the isolation of two flavone derivatives was reported, namely 4′-O-methyl-isoscutellarein (**12**) and 4′-O-methyl-isoscutellarein-7-O-(6″′-O-acetyl)allopyranosyl-(1→2)-glucopyranoside (**21**) [95].

Furthermore, our survey revealed the presence of poly-methylated flavones in the genus *Stachys* (Table 3 and Table 17). Precisely, six species and four subspecies from subgenus *Stachys*, as well as one species from subgenus *Betonica*, are found to contain poly-methylated flavones. The most common representative was xanthomicrol (**69**) which was mentioned in seven *Stachys* species and subspecies of different sections from the subgenus *Stachys* [20,68,74,77,78,102,107]. In the stems of the species *S. schtschegleevii*, apart from xanthomicrol (**69**), was also found circimaritin (**66**) [74].

A few studies mentioned the existence of flavonols in *Stachys* spp. (Table 4 and Table 18), mainly in species occurred in Greece. Afouxenidi and colleagues (2018) isolated kaempferol (**91**) from the *n*-butanol residue of the aerial parts of *S. tetragona* [100], which was also identified in the aerial parts of *S. cretica* subsp. *smyrnaea* [81]. Moreover, isorhamnetin (**92**) was isolated from the methanol extract of the aerial parts of *S. swainsonii* subsp. *swainsonii* and *S. swainsonii* subsp. *argolica* [102]. A study conducted by Marin et al. (2004) identified the presence of quercetin 3-O-rutinoside (**93**) and isorhamnetin 3-O-glucoside (**94**) from the aerial parts of *S. palustris* [5].

In addition, three flavanones were isolated from three species of the genus *Stachys* (Table 5 and Table 19). Eriodictyol (**95**) was mentioned in *S. cretica* [108] and in one subspecies of *S. swainsonii* [102], while naringenin (**96**) was isolated from the aerial parts of the species *S. aegyptiaca* [104]. A flavanone rutinoside, known as hesperidin (**97**), was identified as one of the major compounds of the aerial parts of *S. cretica* subsp. *smyrnaea* [81].

Of great interest is the isolation of a rare diflavone ester of *μ*-truxinic acid, namely stachysetin (**98**). It is well-known that diglycoside flavone esters of dicarboxylic acids are rare compounds in plant kingdom. Stachysetin was firstly isolated from the ethanol extract (70% v/v) of the aerial parts of *S. aegyptiaca* [69]. Then, Murata and co-workers (2008) reported it in the methanol residue (80% v/v) of the aerial parts of *S. lanata* [82]. In a current study carried out by Pritsas et al. (2020), stachysetin was isolated from the methanol: aqueous (5:1) extract from the flowering aerial parts of the cultivated *S. iva* (Table 6 and Table 20) [56]. Up to now, there is no report of this secondary metabolite in the species of the subgenus *Betonica*. The presence of this rare natural compound in the sections Ambleia, Eriostomum and Candida of the subgenus *Stachys* might be considered as a chemotaxonomic marker among the two subgenera and of the genus *Stachys*.

### 4.2. Phenolic Derivatives; Acetophenone Derivatives

Regarding the phenolic derivatives of genus *Stachys* (Table 7 and Table 21), mainly chlorogenic acid (**103**) was appeared in nine *Stachys* species; *S. candida* [78], *S. iva* [56], *S. cretica* (*S. cretica* subsp. *smyrnaea* [81], *S. cretica* subsp. *mersinaea* [108], *S. cretica* subsp. *vacillans* [112]), *S. lanata* [82], *S. tmolea* [85], *S. thirkei* [84], *S. recta* [14], *S. palustris* [104] and *S. officinalis* [111]. The isomers of chlorogenic acid (**102**, **104**, **105**) also reported in *S. atherocalyx* [110], *S. recta* [14] and *S. palustris* [23,104]. Caffeic (**108**) and *p*-coumaric (**106**) acids were found in two *Stachys* spp. [104,110]. Moreover, Kirkan (2019) identified vanillic (**100**) and syringic (**101**) acids from the aerial parts of *S. cretica* subsp. *vacillans* [112]. Though, 4-hydroxybenzoic acid (**99**) was reported from *S. tmolea* [85]. Arbutin (**107**) was also identified in the aerial parts of *S. germanica* subsp. *salviifolia* [109]. One study also reported the presence of acetophenone derivatives from the roots of *S. lanata*, namely androsin (**109**), neolloydosin (**110**) and glucoacetosyringone (**111**) (Table 8 and Table 22) [82]. The isolation of the latter compounds might be attributed to the different investigated plant parts (roots).

### 4.3. Lignans

Lignans are types of polyphenols with diverse structures. Although these bioactive compounds were presented in Lamiaceae family [149], a few studies reported their existence in plants of genus *Stachys*. Specifically, three lignans categorizing into two furanofuran-type derivatives (sesamin and paulownin) and one benzofuran-type lignan (urolignoside) were reported in two species of the subgenus *Stachys* (Table 9 and Table 23). Laggoune et al. (2016) isolated sesamin (**112**) and paulownin (**113**) from the aerial parts of *S. mialhesii* [103], while urolignoside (**114**) was isolated from the aerial parts of *S. tetragona* [100]. Given that up to now there is no study reported the presence of lignans in the subgenus *Betonica*, the identification of lignans might be considered as a chemotaxonomic difference between the two subgenera *Stachys* and *Betonica*.

### 4.4. Phenylethanoid Glycosides; Phenylpropanoid Glucosides

The present review unveiled 29 phenylethanoid glycosides in 17 *Stachys* species (Table 10 and Table 24). Acteoside or verbascoside (**118**) was the most abundant found in 16 *Stachys* spp. of all sections through this survey. Additional phenylethanoid glycosides isolated and identified from this genus includes martynoside, leucosceptoside A and lavandulifoliosides. Lavandulifolioside A (or stachysoside A) (**129**) was firstly isolated from the methanol extract of the aerial parts of *S. lavandulifolia* in 1988 [115], while in 2011 Delazar et al. (2011) isolated lavandulifolioside B (**130**) from the same plant, for the first time [12]. Moreover, three phenylethanoid glycosides were reported from the aerial parts of *S. byzantina* (section Eriostomum), including verbascoside (**118**), 2′-O-arabinosyl verbascoside (**122**) and aeschynanthoside C (**133**) [35]. Among them, the first and the last compound has been isolated only from the specific species. A survey conducted by Murata and co-workers (2008) reported ten phenylethanoid glycosides from different plant parts [82]. In the aforementioned study, leonoside B (or stachysoside D) (**134**) and martynoside (**135**) were mentioned from the aerial parts of *S. lanata*, while from the roots of the specific species were reported eight phenylethanoid glycosides, namely rhodioloside (**115**), verbasoside (**116**), 2-phenylethyl-D-xylopyranosyl-(1→6)-D-glucopyranoside (**117**), verbascoside (**118**), isoacteoside (**119**), darendoside B (**120**), campneoside II (**121**) and campneoside I (**136**). It is remarkable to point out that compounds **115**, **117** and **120** haven′t been reported in other *Stachys* species. This might be attributed to the fact that the plant material was roots. Another study carried out by Karioti et al. (2010) focused on the phenolic compounds from the aerial parts of *S. recta*, and reported many phenylethanoid glycosides from its aerial parts, including acteoside (**118**), isoacteoside (**119**), β-OH-acteoside (**121**), betunyoside E (**127**), campneoside I (**136**), forsythoside B (**137**), β-OH-forsythoside B methyl ether (**138**) [14]. Furthermore, lamiophloside A (**141**) was isolated with some other phenylethanoid glycosides from the aerial parts of *S. tetragona* [100]. Of great interest is that our survey revealed that this constituent is mentioned only in the specific species. Two rare phenylethanoid glycosides, parviflorosides A-B (**142**–**143**) were isolated from the whole plant of *S. parviflora* [120]. These two compounds are characterised by the presence of a third saccharide (rhamnose) linked to the proton H-2′ of glucose, comparing to others common phenylethanoid glycosides where the connection of the third saccharide is in proton H-3′ of glucose. Of great interest is that *S. parviflora* is now considered as the monotypic genus *Phlomidoschema* (only *P. parviflorum* (Benth.) Vved.) [2]. Furthermore, leonoside A (or stachysoside B) (**139**) was isolated with other three phenylethanoid glucosides from the whole plant of *S. riederi* [114]. To be mentioned that phenylethanoid glycosides were reported in both subgenera of genus *Stachys*.

Apart from phenylethanoid glucosides, Murata et al. (2008) mentioned two phenylpropanoid glucosides in the roots of *S. lanata* (subg. *Stachys*; sect. Eriostomum), coniferin (**144**) and syringin (**145**) (Table 11 and Table 25) [82]. It is worth to mention that the isolation of phenylpropanoid glucosides only from the specific plant, might be assigned to the different studied plant material (roots).

### 4.5. Iridoids

Iridoids are among the major chemical compounds found in genus *Stachys*. According to Tundis et al. (2014), iridoids are considered as good chemotaxonomic markers of this genus [3]. Accumulating phytochemical studies have reported diverse types of iridoids [3]. The present review summarises all these studies, exemplifying 38 *Stachys* species which their iridoid cargo has been investigated (Table 12 and Table 26). Harpagide (**148**; 31 species) and its acetyl derivative; 8 acetyl-harpagide (**150**; 28 species) are of common occurrence in genus *Stachys* and might be considered as characteristic iridoids of these plants. Furthermore, ajugol (**146**; 18 species), ajugoside (**147**; 18 species), melittoside (**166**; 17 species), monomelittoside (**165**; 4 species) and 5-allosyloxy-aucubin or 5-O-allopyranosyl-monomelittoside (**167**; 4 species/1 subsp.) were also mentioned in various species. Allobetonicoside (**161**) was firstly isolated from the aerial parts of *S. officinalis* [127] and then from the aerial parts of *S. glutinosa* [122] and of *S. macrantha* [117]. The latter study also mentioned the isolation of cinnamoyl-harpagide derivative, macranthoside (**156**), for the first time. To be mentioned that Jeker et al. (1989) also isolated 6-O-acetylmioporoside (**155**) from the aerial parts of *S. officinalis* [127]. In addition, two species revealed the presence of 8-*epi*-loganic acid (**157**), 8-*epi*-loganin (**159**) and gardoside (**160**) [20,56], as well as 7-O-acetyl-8-*epi*-loganic acid (**158**) was only mentioned from the aerial parts of *S. spinosa* [98]. Of note, Iannuzzi et al. (2019) isolated from the leaves of *S. ocymastrum* (syn. *S. hirta* L.) five iridoids which haven′t been documented in other species, namely 6β-acetoxyipolamiide (**172**) 6β-hydroxyipolamiide (**173**), ipolamiide (**174**), ipolamiidoside (**175**) and lamiide (**176**) [123]. A study conducted by Háznagy-Radnai (2006) examined the phytochemical profiles of *Stachys* spp. growing in Hungary, reporting the iridoid content of ten taxa [124]. Murata and co-workers (2008) isolated five new esters of monomelittoside from the aerial parts and roots of *S. lanata* [82]. In particular, stachysosides E (**168**), G-H (**170–171**) were found in roots, while stachysosides E (**168**) and F (**169**) were discovered from the aerial parts of the specific species. It is important to be mentioned the detection of a new iridoid diglycoside, 4′-O-β-D-galactopyranosyl-teuhircoside (**162**), which was isolated from the flowering aerial parts of *S. alopecuros* subsp. *divulsa* [119]. Muñoz et al. (2001) reported the presence of 5-desoxy-harpagide (**151**) and 5-desoxy-8-acetyl-harpagide (**152**) from the aerial parts of *S. grandidentata* [129]. Notably, this review unveiled some differences in iridoids among subgenera *Stachys* and *Betonica*. Firstly, it was observed that there is no report for the presence of monomelittoside or melittoside derivatives in the subgenus *Betonica*. Secondly, reptoside (**153**) was found in two species of subgenus *Betonica* (*S. macrantha* and *S. officinalis*) and not in the plants of subgenus *Stachys*.

### 4.6. Diterpenes

A landmark study for diterpenes of genus *Stachys* is the review article of Piozzi and Bruno (2011), including all the reported diterpenoids from roots and aerial parts of *Stachys* spp [21]. Up to now, several types of diterpenes have been mentioned, comprising types of *neo*-clerodane, labdane, rosane and *ent*-kaurene skeleton (Table 13 and Table 27). The most common type is the *neo*-clerodane skeleton derivates, as they were found in various species. *S. aegyptiaca* has thoroughly studied for its phytochemical profile. A study conducted by Hegazy et al. (2017) reported the discovery of three new *neo*-clerodane diterpenoids from the aerial parts of the aforementioned plant, namely stachaegyptins A-C (**190**–**192**) [106]. One year later, two new compounds were mentioned; stachaegyptins D-E (**193**–**194**) [131], while in a current work stachaegyptins F-H were isolated (**195**–**197**) [133]. Moreover, stachysperoxide (**189**) was isolated from the *S. aegyptiaca* [132]. These stachaegyptin derivatives and stachysperoxide reported only in the species *S. aegyptiaca* and might be a characteristic chemical compound of the specific plant of the section Ambleia. Derkach (1998) mentioned the compounds annuanone (*cis*-clerodane type) (**181**), stachylone (**182**) and stachone (**183**) in five species of the subgenus *Stachys*; *S. atherocalyx*, *S. inflata*, *S. iberica* and *S. sylvatica* [134]. Other *neo*-clerodane type diterpenes which were found in many species are roseostachenone (**184**), roseostachone (**185**), roseostachenol (**186**) and roseotetrol (**187**). Ruiu and co-workers (2015) explored the aerial parts of *S. glutinosa*, isolating roseostachenone and the new *neo*-clerodane diterpene, 3α,4α-epoxyroseostachenol (**188**) [107]. Furthermore, labdane type derivatives were occurred in the genus *Stachys*. Fazio et al. (1994) investigated the aerial parts of *S. mucronata* and isolated three labdane skeleton compounds; ribenone (**198**), ribenol (**199**) and 13-*epi*-sclareol (**200**) [57]. The latter compound has also been found in *S. rosea* [141]. Paternostro et al. (2000) studied the aerial parts of *S. plumosa*, determining the following labdane type derivatives (+)-6-deoxyandalusol (**201**), 13-*epi*-jabugodiol (**202**) and (+)-plumosol (**203**) [144]. The compound (+)-6-deoxyandalusol were also found in *S. distans* and *S. ionica* [139]. Some *ent*-kaurene derivatives were reported in *S. aegyptiaca* [130], *S. lanata* [135] and *S. sylvatica* [142]. Moreover, one abietane diterpenoid, horminone (**211**), was isolated from the aerial parts of *S. mialhesii* [103]. It is noteworthy to be underlay the presence of two rare rosane type diterpenes in the aerial parts of *S. parviflora*, namely stachyrosanes 1 (**212**) and 2 (**213**) [134]. In addition, six diterpene lactone derivatives, i.e., betolide (**214**), betonicolide (**215**) and betonicosides A-D (**216**–**219**) were found in the species *S. officinalis* [143,145] and *S. scardica* [143] of the subgenus *Betonica*.

In the context of chemotaxonomic significance, it could be observed that species of subgenus *Stachys* product mainly *neo*-clerodane and labdane type derivatives, while the plants of subgenus *Betonica* biosynthesized diterpene lactone derivatives. Thus, the latter derivatives might be recognised as characteristic chemotaxonomic markers of subgenus *Betonica*. Another important chemotaxonomic point is reported by Piozzi et al. (2002), mentioning that (+)-6-deoxyandalusol has been determined only in three *Stachys* species of eastern part of the Mediterranean region [139].

### 4.7. Triterpene Derivatives, Phytosterols and Phytoecdysteroids

Triterpene derivatives and phytosterols are major secondary metabolites of Lamiaceae family. In genus *Stachys*, five phytosterol derivatives (**220**–**224**) were found in *S. byzantina* [17,35], *S. annua* [95], *S. spinosa* [99], *S. tetragona* [100], *S. palustris* [146] and *S. alopecuros* subsp. *divulsa* [119] (Table 14 and Table 28). Furthermore, the triterpenoids; ursolic (**226**) and oleanolic (**227**) acids were only reported from the section Olisia (subg. *Stachys*) [95,99,100]. Kotsos et al. (2007) isolated an oleanolic lactone derivative (**228**) of the aerial parts of *S. spinosa* [99]. It is noteworthy to be mentioned the presence of saponin derivatives in genus *Stachys* (Table 14 and Table 28). The first saponins isolated from this genus were from the water extract of the whole plant of *S. riederi*, including 8 stachyssaponins (I-VIII, **231–238**) [147]. Afterwards, stachyssaponins A-B (**229–230**) were found from the methanol extract of the aerial parts of *S. parviflora* [63].

Few *Stachys* spp. include phytoecdysteroids (Table 14 and Table 28). Ramazanov and co-workers (2016) isolated five phytoecdysteroids from *S. hissarica* [67], namely 20-hydroxyecdysone (**239**), polipodin B (**240**), integristeron A (**241**), 2-desoxy-20-hydroxyecdysone (**242**) and 2-desoxyecdyson (**243**).

### 4.8. Other Chemical Categories

Notable among the above-mentioned classes of compounds are the megastigmane derivatives from *Stachys* spp. (Table 15 and Table 29). Takeda and colleagues (1997) isolated from the aerial parts of *S. byzantina* five bioactive compounds from this group, including byzantionosides A-B (**244**,**245**), icariside B2 (**246**), (6R, 9R)- and (6R, 9S)-3-oxo-α-ionol glucosides (**247**) and blumeol C glucoside (**248**) [148]. Furthermore, vomifoliol (**249**) and dehydrovomifoliol (**250**) were reported from the aerial parts of *S. lanata*, while citroside A (**251**) was isolated from the roots of this species [82]. This study also mentioned the presence of sugar ester (cistanoside F) from the roots of *S. lanata* [82]. At this point, we should note that few studies reported some oligosaccharides from *Stachys* spp. [3]. For instance, stachyose is a tetrasaccharide which consists one of the most common oligosaccharides in genus *Stachys* and shows beneficial effects for the gastrointestinal system as it can be directly consumed [3,23,119,150]. Precisely, the species *S. sieboldii* is a major source of this constituent [27,151,152]. Stachyose is an oligosaccharide, which can be directly consumed for the benefit of gastrointestinal system [150]. Furthermore, Yin and colleagues (2006) mentioned that the bitter taste of some *Stachys* species, such as *S. annua* and *S. balansae*, might be attributed to their bitter diterpene derivatives, like stachylone [22,151].

## 5. Pharmacological Activities

This section includes the most interesting pharmacological data of the last five years (from 2015 to 2020). Many studies exemplified the great antimicrobial, antioxidant and cytotoxic effects of the essential oils of these plants [3,15]. Tundis et al. (2014) described in detail the biological studies (in vitro and in vivo) of the essential oils, extracts and compounds [3]. Thus, in the present review, we focused on the current available pharmacological researches of the extracts and isolated compounds from *Stachys* spp. as they are presented in Table 30.

### 5.1. Antioxidant Activity/Cytoprotective

Tundis et al. (2015) evaluated five extracts (*n*-hexane, dichloromethane, methanol, methanol with Soxhlet apparatus and ethanol 70% extract) from the aerial parts of *S. lavandulifolia* for their antioxidant activity, using β-carotene bleaching test, 2,2′-azino-bis(3-ethylbenzothiazoline-6-sulphonic acid (ABTS), 1,1-Diphenyl-2-picrylhydrazyl (DPPH), and Ferric Reducing Antioxidant Power (FRAP) assays [116]. The most polar extracts, ethanol 70% and methanol extracts, showed the highest radical scavenging activity against ABTS radical (IC_50_ values of 19.9 and 22.8 μg/mL, respectively), whereas the methanol extract Soxhlet apparatus was the most active in the DPPH method (IC_50_ of 25.0 μg/mL). In the β-carotene bleaching test, the methanol and ethanol extract demonstrated the stronger activity after 30 min of incubation (IC_50_ = 29.3 and 33.0 µg/mL, respectively) and the IC_50_ values were of 60.3 and 34.6 µg/mL, respectively after 60 min of incubation. Moreover, they studied the antioxidant activity of bioactive secondary metabolites; arbutin (**107**), acteoside (**118**), monomelittoside (**165**), melittoside (**166**), 5-allosyloxy-aucubin (**167**), and stachysolone (**177**), reporting that in both DPPH and ABTS assays the most active compounds was arbutin (**107**) with IC_50_ values of 62.5 and 45.7 μg/mL, respectively [116]. Another work investigated the antioxidant activity of three extracts of *S. guyoniana*, through β-carotene–linoleic acid, DPPH, ABTS, CUPric Reducing Antioxidant Capacity (CUPRAC) and metal chelating assays [155]. The chloroform extract had the highest antioxidant activity (IC_50_ = 2.3 ± 1.27 μg/mL) in β-carotene–linoleic acid and in ABTS method (IC_50_ = 7.29 ± 0.23 μg/mL). The *n*-butanol extract showed the better antioxidant capacity in DPPH test (IC_50_ = 2.91 ± 0.14 μg/mL) compared to other extracts and to the reference compound α-tocopherol (IC_50_ = 7.31 ± 0.17 μg/mL), as well as it demonstrated highest activity in CUPRAC method (A_0.50_ = 0.15 ± 0.05 μg/mL) and in metal cheating assay (inhibition at 100 μg/mL: 48%). In addition, Laggoune et al. (2016) demonstrated the great antioxidant properties in vivo of *S. mialhesii* [103]. Particularly, the *n*-butanol extract of the specific plant showed IC_50_ value of 0.047 mg/mL in DPPH assay, while the IC_50_ value of the isolated compound isoscutellarein-7-O-[6″′-O-acetyl]-β-D-allopyranosyl-(1→2)-β-D-glucoside (**15**) was 0.066 mg/mL and the reference compound quercetin was 0.012 mg/mL. Notably, they also mentioned that the extract (up to 10 g/kg) did not show any toxicity in mice during 24 h after administration. In addition, the antioxidant activity of three subspecies of *S. cretica* (i.e., *S. cretica* subsp. *mersinaea*; *S. cretica* subsp. *smyrnaea*; *S. cretica* subsp. *vacillans*) were investigated in different works [81,108,112]. The antioxidant capacity of the methanol extract of *S. parviflora* was measured, exhibiting an IC_50_ value of 76.87 ± 0.57 μg/mL (DPPH method) and of 188.47 ± 0.76 μg/mL (β-carotene bleaching test; BCB), while the standard compound, butylated hydroxytoluene (BHT), had stronger activity in both tests (DPPH test: IC_50_ = 1.23 ± 0.02 μg/mL; BCB test: 34.31 ± 0.40 μg/mL) [64]. Guo et al. (2018) examined the antioxidant activity of five fractions from the 70% ethanol extract of tubers of *S. affinis* by DPPH assay and superoxide radical scavenging activity [28]. The ethyl acetate fraction showed extremely high antioxidant activity in DPPH method (IC_50_ = 0.85 ± 0.04 μg/mL) with α-tocopherol as positive control (IC_50_ = 18.68 ± 0.51 μg/mL). They reported that this great antioxidant activity was attributed to the high content in phenolics and flavonoids of this fraction and confirmed the use of this plant as a natural antioxidant. Another work studied the antioxidant activity of the extracts and fractions of the same *Stachys* species on reactive oxygen species (ROS) production induced by H_2_O_2_ in HT-1080 cells [29]. In particular, the *n*-hexane fraction decreased H_2_O_2_-induced ROS and oxidative stress-induced DNA damage, as well as it increased glutathione (GSH) production. The species *S. mucronata* demonstrated strong anti-radical activity due to the high content in polyphenols [156]. A recent study conducted by Aminfar et al. (2019) described a chemometric-based approach in order to classify *S. lanata* by Gas Chromatography-Mass Spectrometry (GC-MS) fingerprints and to correlate their chemical constituents with their antioxidant capacity [35]. They identified eight antioxidant markers which could also serve as volatile markers. In addition, Elfalleh and co-workers (2019) demonstrated the differences of the antioxidant properties of the extracts of *S. tmolea*, reporting that water extract exhibited highest activity than methanol extract, using DPPH, ABTS, CUPRAC, FRAP, phosphomolybdenum and ferrous ion chelating methods [85]. A survey conducted by Hwang et al. (2019) demonstrated that the ethanol extract of *S. riederi* var. *japonica* exhibited antioxidant effects on ultraviolet A (UVA)-irradiated human dermal fibroblasts (HDFs), through suppression of ROS generation [160]. The antioxidant activity of the methanol extract of the Lebanese species *S. ehrenbergii* was measured by ABTS radical cation decolorization assay and the methanol extract showed an IC_50_ value of 52 ± 7.5 mg/mL [154]. Furthermore, the chemical profile and some biological activities of three herbal teas in Anatolia were examined [84]. Among them, the methanol extract of *S. thirkei* showed strongest antioxidant capacity, through β-carotene (IC_50_ = 47.79 ± 0.59 μg/mL), DPPH (IC_50_ = 49.31 ± 0.38 μg/mL), ABTS (IC_50_ = 13.34 ± 0.02 μg/mL) and CUPRAC (absorbance%: 1.88 ± 0.02 μg/mL) assays. Sadeghi et al. (2020) assessed the the antioxidant properties of hydroalcoholic extract of *S. pilifera* on nephrotoxicity induced with cisplatin (CP) in vivo (in rats), showing that the specific extract restored the antioxidant capacity, as well as it had renoprotective activity [19].

### 5.2. Cytotoxicity and Antiproliferative Activity

Venditti et al., (2017) investigated the cytotoxic activity and the anti-reactive oxygen species activity of the ethanol extract from tubers of the Chinese artichock (*S. affinis*) [27]. Regarding the cytotoxicity, the specific extract didn′t demonstrate any activity in K562, SH-SY5Y and Caco-2 cell lines, even at the highest concentrations (1.0 mg/mL). The cytotoxic activity of extracts and isolated flavonoids from the aerial parts of *S. lavandulifolia* were studied by Delnavazi et al. (2018) through the 3-(4,5-dimethylthiazol-2-yl)-2,5-diphenyltetrazolium bromide (MTT) assay [13]. The dichloromethane extract showed the highest cytotoxic activity in brine shrimp lethality test (BSLT) (LD_50_ = 121.8 ± 5.6 μg/mL), while as a positive control was used podophyllotoxin (LD_50_ = 3.1 ± 0.6 μg/mL). Afterwards, they explored the cytotoxic activity of isolated flavonoids in three cancer cell lines (MDA-MB-231, HT-29 and MRC-5), using as reference compound tamoxifen. All the nine isolated flavonoids moderated the cytotoxicity activated on the studied cell lines. However, chrysosplenetin (**84**) was reported as the most active compound in the first two cell lines. In MRC-5 cell line, apigenin (**1**) exhibited the greatest activity. It is remarkable to point out that the specific study also mentioned the selective activity against cancer cells, reporting that chrysosplenetin (**84**), kumatakenin (**79**) and viscosine (**78**) exhibited higher selective toxicity against MDA-MB-231 cell line than tamoxifen. At this point, we should underlie that the great cytotoxic activity of these compounds is attributed to their substitutions with (poly)-methylated groups which increase this effect. Another study evaluated the methanol extract, the alkaloid and the terpenoid fractions of *S. pilifera* for their cytotoxic and antiproliferative activity in vitro (HT-29 cell line), indicating great results [45]. The terpenoid fraction was found to have the best cytotoxic activity compared to the other fractions and as reference compound was used cisplatin. Moreover, they investigated the antiproliferative activity, studying the effects on the activity of caspase-8 and caspase-9, Nuclear factor-κB (NF-κB) and Nitric Oxide (ΝO), reporting that the extract/fractions increased the activity of caspase-8/-9 and decreased NF-κB and subsequently NO level. Of note, they compared their results with previous data of cytotoxic activity in vitro of other *Stachys* species such as *S. acerosa, S. benthamiana, S. floridana, S. lavandulifolia, S. obtusicrena, S. persica, S. pubescens* and *S. spectabilis*. Three isolated compounds from the extract (CH_2_Cl_2_:MeOH 1:1) of the aerial parts of *S. aegyptiaca* were investigated for the cytotoxic activity in HepG2 cell line, using MTT assay [132]. Precisely, the IC_50_ values of stachaegyptin D (**193**), stachysolon monoacetate (**178**) and stachysolon diacetate (**180**) were 94.7, 63.4 and 59.5 μM, respectively, with stachysolone diacetate being the most active. In another study, the cytotoxic effect of the ethanol extract of *S. riederi* var. *japonica* on UVA-irradiated HDFs was evaluated at different concentrations for 48 h by MTT assay, showing no or little cytotoxicity [160]. Shakeri et al. (2019) mentioned that the methanol extract of *S. parviflora* demonstrated no cytotoxic activity toward the cancer cell lines, namely A2780, HCT, and B16F10 in all tested concentrations (>100 µg/mL) [64]. Moreover, the genotoxic activity of the extracts from four different plants were investigated by Slapšytė and colleagues (2019) [157]. They reported that all the plant extracts induced DNA damage, using the comet assay, whereas the extract of *S. officinalis* induced the increase of sister chromatid exchange value. The methanol extract of the Lebanese species *S. ehrenbergii* was investigated for its antioxidant and cytotoxic activity [154]. The cytotoxicity was examined by MTT assay where the methanol extract showed the highest cytotoxicity (IC_50_ = 420 ± 104 μg/mL) at a concentration of 3000 mg/mL.

### 5.3. Polycystic Ovary Syndrome (PCOS)

In Iran, *S. sylvatica* is used for the treatment of women with polycystic ovary syndrome (PCOS). A current study carried out by Alizadeh et al. (2020) evaluated the hydroalcoholic extract of this plant in a rat model of PCOS [47]. It was observed that the extract at the dose of 500 mg/kg increased gonadotropins FSH and LH (5.95 ± 0.02 mIU/mL; 6.48 ± 0.09 mIU/mL) and reduced the level of estrogen (0.9 ± 0.07 mIU/mL) compared to the PCOS group (FSH level: 1.69 ± 0.08 mIU/mL; LH level: 6.29 ± 0.04 mIU/mL; estrogen level: 1.42 ± 0.05 mIU/mL), causing the ratio of LH/FSH to be close to 1:1 (6.48/5.59). According to the literature, this ratio LH/FSH is almost 1:1 in normal cases, while in PCOS women is higher e.g., 2:1 or 3:1. They also mentioned that these great results of the extract of *S. sylvatica* could be correlated to the flavonoid content of the plant. Previous studies showed that flavonoids could decrease the level of estrogen and could also act as GABA receptor agonists, regulating gonadotropins. Given that women with PCOS showed high concentrations of inflammation factors, they assumed that the extract could act as anti-inflammatory and antioxidant agent as flavonoids and iridoids demonstrated antioxidant and anti-inflammatory properties.

### 5.4. Anticholinesterase and Anti-Alzheimer’s Activity/Neuroprotective Activity

The aqueous extract from the tubers of *S. sieboldii* (“chorogi”) was studied in vivo in mice model for its neuroprotective potential [152]. Specifically, the study examined the effects of chorogi’s extract on celebral ischemia and scopolamine-induced memory impairment, using as positive control the extract of *Gingko biloba*, proving that *S. sieboldii* improves the learning and memory dysfunction correlated with ischemic brain injury. Another work examined the cholinesterase inhibitory activity of *S. lavandulifolia* extracts and isolated compounds [116]. Specifically, the most active extract against anticholinesterase (AChE) was the *n*-hexane extract with an IC_50_ value of 13.7 μg/mL. However, the dichloromethane extract was the most effective against butyrylcholinesterase (BChE) (IC_50_ = 143.9 μg/mL) where its major constituent, stachysolone (**177**), inhibited the activity of this enzyme with a percentage of inhibition of 50% at 0.06 mg/mL. Among the studied polar extracts, the methanol extract exhibited a selective inhibitory activity against AChE with an IC_50_ value of 211.4 μg/mL and the isolated compounds, arbutin (**107**) and 5-allosyloxy-aucubin (**167**), showed a percentage of inhibition of 50 and 23.1% at 0.06 mg/mL, respectively, against AChE. Notably, the other constituents of this species were inactive at the maximum concentration tested of 0.25 mg/mL. Ferhat et al. (2016) examined the AChE activity of *n*-butanol, the ethyl acetate and the chloroform extracts of the aerial parts of *S. guyoniana*, demonstrating that the *n*-butanol extract (IC_50_ = 5.78 ± 0.01 μg/mL) was a little less active than the used standard drug against Altzheimer’s disease; galantamine (IC_50_ = 5.01 ± 0.10 μg/mL). Furthermore, they exhibited that this extract inhibited the BChE, having an IC_50_ value of 39.1 ± 1.41 μg/mL which was better than the standard (IC_50_ = 39.10 ± 1.41 μg/mL) [155]. Moreover, the anti-Alzheimer’s activity of two subspecies of *S. cretica* (*S. cretica* subsp. *smyrnaea; S. cretica* subsp. *mersinaea*) were evaluated in different works [81,108]. In addition, the potential effects of 20% ethanol extract of *S. sieboldii* was evaluated against oxidative stress induced by H_2_O_2_ in SK-N-SH cells and memory enhancement in ICR mice [162]. This study showed that the daily intake of the extract (dose: 500 mg/kg) through dietary supplementation produced memory enhancing effects in animals. Recently, Ertas and Yener (2020) reported that the acetone extract of *S. thirkei* demonstrated good activity against AChE and BChE with a percentage of inhibition of 52.46 ± 1.26% and 75.04 ± 1.91%, respectively [84].

### 5.5. Anti-tyrosinase Activity

The anti-tyrosinase activity of the ethanol and methanol Soxhlet apparatus extracts of the aerial parts of *S. lavandulifolia* exhibited the best activity with IC_50_ values of 33.4 ± 0.8 and 42.8 ± 1.1 μg/mL [116]. They underlay that the specific extracts were characterized by the phenolic compounds, acteoside (**118**) and arbutin (**107**), which are recognised as tyosinase inhibitors. Moreover, they evaluated the anti-tyrosinase activity of the isolated iridoids among which monomelittoside (**165**) and melittoside (**166**) showed IC_50_ values of 119.6 ± 2.2 and 163.1 ± 3.1 μg/mL respectively, while 5-allosyloxy-aucubin (**167**) inhibited the enzyme with a percentage of 22.4% at a concentration of 200 μg/mL. In addition, current works investigated the anti-tyrosinase activity of three subspecies of *S. cretica* (*S. cretica* subsp. *smyrnaea; S. cretica* subsp. *mersinaea; S. cretica* subsp. *vacillans*), reporting that the ethyl actetate extract was the most effective in the first two susbspecies (2.45 mg KAEs/g; 16 mg KAEs/g, respectively) [81,108]. Though, the methanol extract of *S. cretica* subsp. *vacillans* had the higher activity against tyrosinase (314.04 ± 2.05 mg KAE/g extract) [112].

### 5.6. Anti-diabetic Activity

Bahadori et al. (2018) evaluated the anti-diabetic activity of the extracts of *S. cretica* subsp. *smyrnaea* [81]. Specifically, the methanol extract demonstrated strong anti-diabetic activity against α-amylase (61.4 mg ACEs/g dry plant) and α-glucosidase (47.8 mg ACEs/g dry plant), following by ethyl acetate extract. They assumed that the above good properties were attributed to the phenolic constituents of the methanol extract since the anti-glucosidase activity is associated with caffeic acid, *trans*-cinnamic acid, and vanillin, whereas the amylase inhibitory activity is related to kaempferol and *p*-hydroxybenzoic acid. A year later, the anti-diabetic activity of the extracts of *S. cretica* subsp. *mersinaea* was studied, reporting that the ethyl acetate extract had best activity against α-amylase (396.50 mgACEs/g), while the methanol extract exerted strong activity against α-glucosidase (734 mg ACEs/g) [108]. Furthermore, the α-amylase inhibition of the methanol and water extract of *S. cretica* subsp. *vacillans* was evaluated, with the methanol extract exhibited stronger activity (433.99 ± 5.10 mg ACE/g extract) [112]. Currently, Pritsas et al. (2020) studied the anti-diabetic activity in silico of 17 isolated compounds from the cultivated *S. iva*, mentioning that stachysetin (**98**) interacted with five out of ten proteins implicated in diabetes [56]. This is the only study reported a pharmacological activity of this rare compound.

### 5.7. Antimicrobial Activity

Regarding the antibacterial activity, the *n*-butanol extract of *S. guyoniana* showed strong activity against *Staphylococcus aureus* (MIC = 32 ± 0.90 μg/mL) and *Enterobacter aerogenes* (MIC = 32 ± 0.70 μg/mL), while it was not active against *Pseudomonas aeruginosa* and *Morganella morganii* [155]. The ethyl acetate extract demonstrated the best inhibition against *Escherichia coli* (MIC = 64 ± 0.60 μg/mL), whereas it didn′t show any activity against *P. aeruginosa* and *M. morganii*. Shakeri et al. (2019) reported the antimicrobial activity of the methanol extract of the aerial parts of *S. parviflora* which exerted the highest activity against the Gram-positive bacterium, *Bacillus cereus*, with a MIC of 0.12 mg/mL [64]. Furthermore, the antimicrobial activity of extracts of *S. thirkei* against different microorganisms were studied according to inhibition zone diameter and MIC value [84]. The acetone and methanol extract demonstrated good activity against *S. aureus*, *Streptococcus pyogenes* and *E. coli*. Intriguingly, *S. thirkeis*′ extracts were not active against *P. aeruginosa* (Gram-negative bacterium) and *Candida albicans* (yeast).

### 5.8. Hepatoprotective

The hepatoprotective property of the ethanol extract of *S. pilifera* was studied in carbon tetrachloride (CCl_4_)-induced hepatotoxicity in rats and indicated that this extract could act as hepatoprotective agent [158]. They assumed that this property might be also related to the strong antioxidant activity of the species. Later, Mansourian et al. (2019) exhibited the hepatoprotective and antioxidant activity of hydroalcoholic extract of *S. pilifera* on hepatotoxicity induced by acetaminophen (APAP) in male rats [159]. Precisely, the extract reduced hepatotoxicity by decreasing liver function markers/enzymes, aspartate aminotransferase (AST) and alanine aminotransferase (ALT) and protein carbonyl (PCO) compared to the APAP group. It also diminished the oxidative stress through inhibiting protein oxidation and inducing the activity of glutathione peroxidase (GPX) enzyme. So, they assumed that this great activity was attributed to the antioxidant activity of this plant.

### 5.9. Others

Ruiu et al. (2015) investigated the phytochemical profile of the dichloromethane extract of *S. glutinosa* and studied the binding affinity to μ and δ opioid receptors (MOR and DOR) [107]. The extract showed an interesting binding affinity for MOR (Ki values of 10.3 μg/mL) and DOR (Ki values of 9.0 μg/mL), while xanthomicrol (**69**) demonstrated the strongest opioid binding affinity to both opioid receptors (Ki for MOR = 0.83 μM, Ki for DOR = 3.6 μM) with the highest MOR selectivity with a ratio Ki (DOR)/Ki (MOR) = 4.4. Notably, they reported that the existence of a further hydroxy group at the 3′ position like in sideritoflavone (**70**) reduced the binding affinity for MOR (Ki = 18.5 μM), whereas the replacement of this group with a methoxy moiety, as in 8-methoxycirsilineol (**71**), eliminated the affinity for MOR (Ki > 50 μM). Furthermore, they evaluated the antinociceptive activity of xanthomicrol in an animal model (in mice) of acute pain (tail-flick test). In another study, the *n*-butanol extract of *S. mialhesii* exhibited significant anti-inflammatory activity *in vivo*, reducing the weight of edema: 52.03% induced by carrageenan in the rat’s paw, whereas indomethacin (dose: 5 mg/kg; decrease 83.36%) was used as a reference drug [103]. In the same study, the *n*-butanol extract exerted antinociceptive effect at dose-dependent manner. Ramazanov et al. (2016) evaluated the wound healing activity of the extract of *S. hissarica* on rats, showing that the extract improved the healing process of linear skin wounds at an oral dose of 10 mg/kg [67]. Of note, the wound healing activity of the extract was more effective than the known drug methyluracil (2,4-dioxo-6-methyl-1,2,3,4- tetrahydropyrimidine), especially in case of alloxan induced diabetic animals. A study carried out by Iannuzzi et al. (2019) studied the antiangiogenic activity in two in vivo models (zebrafish embryos and chick chorioallantoic membrane assays) of the isolated compounds of the leaf extract of *S. ocymastrum*. The isolated compounds with the best antiangiogenic activity in both assays were β-hydroxyipolamiide (**173**) and ipolamiide (**174**) [123]. Recently, Lee et al. (2020) studied the anti-obesity and anti-dyslipidemic property of the roots powder of *S. sieboldii* in rats, following a high-fat and high-cholesterol diet (HFC) [161]. This powder demonstrated the anti-adipogenic and lipid-lowering effects through enhancing lipid metabolism.

Taken together all the above pharmacological studies, we could observe that these findings confirmed most of the traditional medicinal uses of *Stachys* spp. However, the present review unveiled that there are still species pharmacologically uncharted.

## 6. Clinical Studies

Through our literature survey, four clinical studies for the species *S. lavandulifolia* were revealed. The first clinical study carried out by Rahzani et al. (2013) reported the effects of the aqueous extract of the specific plant (dose; infusion from 3 g aerial parts of plant, twice daily) on the oxidative stress in 26 healthy humans, underlying that the participants demonstrated a significant reduction in oxidative stress [163]. In parallel, another randomized clinical trial (33 women) examined the effects of *S. lavandulifolia* and medroxyprogesterone acetate (MPA) in abnormal uterine bleeding (AUB) in PCOS [164]. This study exemplified that the infusion of the aerial parts of wood betony (dose; 5 g of plant in 100 mL boiling water; duration 3 months) showed a reduction of AUB, recommending its consumption for the treatment of AUB related to PCOS. They also mentioned that this result might be attributed to the flavonoid content of the plant and mainly to apigenin. In addition, Monji et al. (2018) evaluated on a clinical trial the therapeutic effects of standardized formulation of *S. lavandulifolia* on primary dysmenorrhea, indicating that the standardized capsules of plant’s extract could diminuish the menstrual pain, and might be recommended as an auxiliary therapy or an alternative remedy to nonsteroidal antiinflammatory drugs (NSAIDs) with fewer side effects in primary dysmenorrhea [165]. Recently, a double-blind randomized clinical study mentioned the analgesic activity of the herbal tea of *S. lavandulifolia* (10 g in 200 cc of boiling water) in 50 patients with migraine [166], showing the capability of this herbal tisane to decrease and also improve the pain intensity in these patients. In addition, Ashtiani et al. (2019) considered that the therapeutic properties of this plant associated with its rich phytochemical profile which include iridoids, flavonoids and phenylethanoid glucosides [166].

To sum up, the above clinical studies confirm the ethnomedicinal uses of *S. lavandulifolia* as a traditional medicine. Although these promising results, more clinical studies should be performed for obtaining data for diverse *Stachys* spp. As a future prospective, further studies should strengthen the research of bioavailability, dosage, toxicity and potential drug interactions in order to endorse the observed pharmacological activities of these plants.

## 7. Toxicity

*S. lavandulifolia* is popularly claimed as an abortifacient agent by Iranian women. The effect of its hydroalcoholic extract on fertility was investigated, revealing that the extract had a dose dependent abortifacient activity. Thus, its use during pregnancy may cause abortion and consequently, the plant should be considered as contraindicated or be used with caution [167]. In addition, the nephrotoxicity of the same extract was studied on male Wistar rats and a mild degeneration of renal tubular epithelial cell after one month was observed, while in the second month the histologic lesions were significantly more. However, further studies need to evaluate renal complications of this plant in human [168]. Moreover, the acute and subchronic toxicological evaluation of *S. lavandulifolia* aqueous extract in rats indicated that the high dose (2 g/kg) did not produce any symptoms of toxicity and there was no significant difference in body weights between the control and treatment groups of the animals [169].

## 8. Conclusions

In the present review, we attempted to describe in detail all the current knowledge and research advances of genus *Stachys*, focusing on pointing the significance of this genus as herbal supplement and medicine.

Taken together with all the analyzed studies in the current review, we categorized the used literature data into four categories according to their general characteristics; ethnobotanical (no of used studies: 48), phytochemical (no of used studies: 91), pharmacological (no of in vitro studies: 22, no of in vivo studies: 8 and 2 in silico study), clinical studies (no of used studies: 4) and reviews (no of used studies: 4). The general characteristics of the analyzed studies in the current review are showed in Table 31.

Several *Stachys* spp. have been used as traditional herbal medicines for thousands of years. Therefore, accumulating studies have been performed in order to explore the chemical compounds and the pharmacological properties of these species to validate their claimed ethnomedicinal properties. However, the present review data shows that there are still species phytochemically and pharmacologically unexplored. This comprehensive survey could serve as useful tool for scientists searching uncharted and interesting species to study, as well as it could be an informative guide for researchers aimed to identify leads for developing novel drugs. Although many pharmacological studies have demonstrated the great properties of these plants, only the clinical effects of one species have been investigated. As a result, further studies should be performed to validate the clinical efficiency of several *Stachys* spp. and if there is any potential toxicity. To be mentioned that there are still yet much to be done on the detailed documentation (safety and efficacy data) of genus *Stachys* in order to be developed an official monograph as a traditional use or well-established use plants.

## Figures and Tables

**Table 1:** *Stachys* species with reported traditional medicinal uses.

Species	Geographical Origin of the Reported Traditional Use	Traditional Medicinal Use	Preparation and/or Administration/ Parts of the Plant	Ref.
*S. acerosa* Boiss.	Iran	Common cold	Decoction	[31]
*S. affinis* Bunge (=*S. sieboldii* Miq.)	China	Infections, colds, heart diseases, tuberculosis, pneumonia	Edible food (tubers)	[27,28]
	China	Common cold, heart diseases, for pain relief, as antioxidant, to treat ischemic brain injury, dementia, various gastrointestinal related diseases	-	[29]
*S. annua* (L.) L	Italy	Headache	Infusion of leaves; also, external use to wash face	[51]
*S. annua* (L.) L subsp. *annua*	Italy	Anti-catarrhal, febrifuge, tonic, vulnerary, against evil eye	Aerial parts	[52]
*S. arvensis* (L.) L.	-	Against evil eye	-	[55]
*S. balansae* Boiss. & Kotschy	-	Hypotonic diseases, cardiac neuroses	Liquid and alcoholic extracts	[23]
*S. byzantina* K. Koch.	-	Anti-inflammatory, antitumor, anticancer, antispasmodic, sedative and diuretic agent, and in the treatment of digestive disorders, wounds, infections, asthma, rheumatic and inflammatory disorders, dysentery, epilepsy, common cold and neuropathy	-	[33]
	Iran	Infected wounds, cutting	Decoction, Demulcent (Leaves)	[34,35]
	Brazil	Antiinflammatory	Infusion of leaves	[60]
*S. cretica* subsp. *anatolica* Rech. f.	Turkey	Colds, stomach ailments	Infusion, decoction, internal	[49]
*S. cretica* L. subsp. *mersinaea* (Boiss.) Rech. f.	Turkey	Colds, stomach ailments	Infusion, decoction, internal	[49]
*S. fruticulosa* M. Bieb.	Iran	Anti- inflammatory	Aerial parts	[32]
*S. geobombycis* C.Y.Wu	China, Japan and Europe	Tonic	-	[22]
*S. germanica* L.	Iran	Gastrodynia, for painful menstruation	Infusion of flowers	[34]
	-	Skin disorders (Veterinary use)	-	[55]
*S. glutinosa* L.	-	As antispasmoic and against chicken louse	-	[55]
*S. iberica* subsp. *georgica* Rech. f.	Turkey	Colds, antipyretic	Decoction, internal	[49]
*S. iberica* subsp. *stenostachya* (Boiss.) Rech. f.	Turkey	Colds, antipyretic, stomach ache	Decoction, internal	[49]
*S. inflata* Benth.	Iran	Infections, asthmatic, rheumatic, inflammatory disorders	Extracts of aerial parts (non flowering stems)	[36,37]
	Iran	Common cold, Analgesic, high blood pressure	Decoction of aerial parts	[31]
*S. iva* Griseb.	Greece	Common cold and gastrointestinal disorders	Decoction, infusion	[56]
*S. kurdica* Boiss & Hohen var. *kurdica*	Turkey	Cold, stomach-ache	Decoction of branches/flowers Drink one glass of the plant on an empty stomach in the morning	[50]
*S. lavandulifolia* Vahl.	Iran	Treat pain and inflammation	Boiled extracts of the aerial parts	[12]
	Iran	Sedative, gastrotonic and spasmolytic properties, treatment of some gastrointestinal disorders, colds and flu	Herbal tea of flowering aerial parts	[13]
	Iran	Headache, renal calculus common cold, sedative flavoring agent, abdominal pain	Decoction of aerial parts, Food additive (aerial parts)	[31]
	Turkey	Antipyretic, cough	Decoction, internal	[49]
	Iran	Painful and inflammatory disorders	Boiled extracts of aerial parts	[41]
	Iran	Anxiolytic influence	Herbal tea	[38,39,40,41,42,43,44]
*S. mucronata* Sieb.	Greece	Antirheumatic and antineuralgic remedy	Decoction for massage	[57]
		For wounds and ulcers	Washed with the decoction and covered with a poultice of fresh leaves for cicatrization	
		Antidiarrhoic agent	Infusion of fresh leaves	
		Pugative	Infusion of roots	
*S. obliqua* Waldst. & Kit.	Turkey	Cold, stomach ailments, fever and cough	Herb, infusion, decoction	[22]
*S. officinalis* (L.) Trevisan (=*S. betonica* Benth.; *Betonica officinalis* L.)	Serbia, Egypt, Montenegro	Skin disorders, antibacterial purposes, against headache, nervous tension, anxiety, menopausal problems, as a tobacco snuff	Tea of dried leaves	[22]
	Italy	Dye wool yellow	Plant	[51]
	Italy	Wounds, in the sores of pack animals	Oily extract of flowers	[54]
*S. palustris* L.	-	Disinfectant, anti-spasmodic and for treatment of wounds	-	[17,61]
	Poland	Wounds, additive in food	-	[58]
	-	Antiseptic, to relieve gout, to stop haemorrhage	-	[62]
*S. parviflora* Benth. (=*Phlomidoschema* *parviflorum* (Benth.) Vved.)	-	Cramps, arthralgia, epilepsy, falling sickness, dracunculiasis	-	[63,64]
*S. pilifera* Benth.	Iran	Toothache, edible, tonic, analgesic, edema, expectorant, tussive	Decoction of aerial parts	[31]
	Iran	Asthma, rheumatoid arthritis and infections	-	[45]
*S. pumila* Banks & Sol.	Anatolia	Antibacterial and healing effects	Tea of the whole part	[21]
	Anatolia	Sedative, antispasmodic, diuretic and emmenagogic properties	Tea of the leaves	[21]
	-	Bronchitis, asthma, stomach pain and gall and liver disorders	-	[65]
*S. recta* L.	Europe	Anxiolytic properties	Herbal tea, Oral administration	[11]
	Italy	Headache	Infusion of leaves to wash face	[51]
	Italy	Bad influence/spirit	Decoction	[53]
	Italy	Depurative	Decoction of the aerial parts	[54]
*S. recta* L. subsp. *recta*	Italy	Tootache and other pain	Aerial parts applied in body parts	[53]
		against anxiety, pain and toothache	Decoction of flowering tops for bath or to wash face, hands and wrists for 3 days	
*S. schtschegleevii* Sosn. ex Grossh.	Iran	Antiinflamatory	Aerial parts	[32,34]
	Iran	Infectious diseases of the respiratoy tract (for colds and sinusitis), for asthma, rheumatism and other inflammatory disorders	-	[46]
*S. sieboldii* Miq. (=*S. affinis* Bunge)	China	Cold and against infections, promoting blood circulation	Dried whole plant	[30]
*S. sylvatica* L.	-	Disinfectant, anti-spasmodic and for treatment of wounds	-	[17]
	Iran	Diuretic, digestive, emmenagogue, antispasmodic, anti-inflammatory, sedative, tonic properties and for the treatment of women with PCOS	-	[47]
	Turkey	Cardiac disorders	Infusion of aerial parts	[48]
*S. tibetica* Vatke	India	For fever, cough, phobias and various mental disorder	Whole plant is boiled and made into a decoction. Drink one teacup decoction twice a day to treat fever for 5–7 days	[66]
*S. turcomanica* Trautv.	Iran	Foot inflammation, toothache, bronchitis and common cold	Infusion, Demulcent, Vapor (Whole plant)	[34]

**Table 2:** Flavones isolated from *Stachys* spp.

Species	Plant Parts	Compound	Ref
Subgenus *Stachys*			
Section Ambleia			
*S. aegyptiaca* Pers.	Aerial parts	Apigenin (**1**), Apigenin 7-O-β-D-glucoside (cosmoside) (**2**), Apigenin 7-O-[6′″-O-acetyl]-allosyl-(1→2)-β-D-glucoside (**3**), Apigenin 6,8-di-*C*-glucoside (Vicenin-2) (**10**), Isoscutellarein 7-O-allosyl-(1→2)-β-D-glucoside (**13**), Isoscutellarein-7-O-[6′″-O-acetyl]-β-D-allopyranosyl-(1→2)-β-D-glucoside (**15**), Luteolin (**34**), Luteolin-7-O-[6′″-O-acetyl]-allosyl]-(1→2)-β-D-glucoside (**39**), 6,8 Di-*C*-β-D-glucopyranosyl luteolin (Lucenin-2) (**40**), Chrysoeriol (**42**) Chrysoeriol 7-O-β-D-glucoside (**43**), Hypolaetin 7-O-[6′″-O-acetyl]-allosyl-(1→2)-[3″-O-acetyl]- β-D-glucoside (**54**), Apigenin 7-O-diglucoside (not determined), Luteolin 7-O-diglucoside (not determined)	[68]
	Aerial parts	Apigenin-7-(3″-E-*p*-coumaroyl)-β-D-glucoside (**4**), Apigenin 7-(6″-*p*-coumaroyl)-β-D-glucoside (**6**)	[69]
	Aerial parts	Isoscutellarein (**11**), 3′,4′-Dimethyl-luteolin-7-O-β-D-glucoside (**41**)	[70]
		Isoscutellarein 8-O-(6″-trans-*p*-coumaroyl)-β-D-glucoside (**18**)	[71]
*S. inflata* Beth.		Scutellarein 7-O-β-D-mannopyranosyl-(1→2)-β-D-glucoside (stachyflaside) (**31**)	[72]
		Isoscutellarein (**11**), 4′-Μethyl-isoscutellarein (**12**), Scutellarein (**29**)	[73]
*S. schtschegleevii* Sosn. ex Grossh.	Stems	Apigenin 7-O-β-D-glucoside (**2**), Apigenin 7-(6″-E-*p*-coumaroyl)-β-D-glucopyranoside (**6**), 3′-Hydroxy-isoscutellarein-7-O-[6′″-O-acetyl]-β-D-glucopyranoside (**14**), Chrysoeriol 7-(6″-E-*p*-coumaroyl)-β-D-glucopyranoside (**47**)	[74]
Section Campanistrum			
*S. arvensis* (L.) L.	Aerial parts ^#^	8-Hydroxyflavone-allosylglucosides (not determined)	[75]
*S. ocymastrum* (L.) Briq. (= *S. hirta* L.)	Aerial parts ^#^	8-Hydroxyflavone-allosylglucosides (not determined)	[75]
	Aerial parts	Apigenin (**1**), Apigenin 7-(6″-E-*p*-coumaroyl)-β-D-glucopyranoside (**6**), Isoscutellarein 7-O-allosyl-(1→2)- glucopyranoside (**13**), Luteolin (**34**)	[76]
Section Candida			
*S. candida* Bory & Chaubard	Aerial parts	Chrysoeriol (**42**), Chrysoeriol 7-(3″-E-*p*-coumaroyl)-β-D-glucopyranoside (**46**)	[77]
	Aerial parts	Apigenin 7-O-β-D- glucopyranoside (**2**), Isoscutellarein 7-O-[6′″-O-acetyl]-β-D-allopyranosyl-(1→2)-β-D-glucopyranoside (**15**), Isoscutellarein 7-O-[6′″-O-acetyl]-allosyl-(1→2)-[6″-O-acetyl]- glucopyranoside (**17**), 4′-Methyl-isoscutellarein 7-O-β-D-[6′″-O-acetyl]-allopyranosyl-(1→2)-β-D-glucopyranoside (**21**), Chrysoeriol 7-O-β-D- glucopyranoside (**43**), Chrysoeriol 7-(3″-E-*p*-coumaroyl)-β-D-glucopyranoside (**46**), 4′-Methyl-hypolaetin-7-O-[6′″-O-acetyl]-β-D-allopyranosyl-(1→2)-β-D-glucopyranoside (**56**)	[78]
*S. chrysantha* Boiss. and Heldr.	Aerial parts	Isoscutellarein 7-O-[6′″-O-acetyl]-allosyl(1→2)-[6″-O-acetyl]-glucoside (**17**), Luteolin 7-O-β-D-glucoside (**37**), Chrysoeriol (**42**), Chrysoeriol 7-O-β-D- glucopyranoside (**43**), Chrysoeriol 7-(3″-E-p-coumaroyl)-β-D-glucopyranoside (**46**)	[77]
*S. iva* Griseb.	Flowering aerial parts	Apigenin (**1**), Isoscutellarein 7-O-[6′″-O-acetyl]-β-D-allopyranosyl-(1→2)-β-D-glucopyranoside (**15**), Isoscutellarein 7-O-[6′″-O-acetyl]-β-D-allopyranosyl-(1→2)-[6″-O-acetyl]-β-D-glucopyranoside (**17**), 4′-Methyl-isoscutellarein 7-O-β-D-[6′″-O-acetyl]-allopyranosyl-(1→2)-β-D-glucopyranoside (**21**), 4′-Methyl-hypolaetin-7-O-[6′″-O-acetyl]-β-D-allopyranosyl-(1→2)-β-D-glucopyranoside (**56**)	[56]
Section Corsica			
*S. corsica* Pers.		Isoscutellarein 7-O-[6′″-O-acetyl]-β-D-allopyranosyl-(1→2)-β-D-glucopyranoside (**15**), 4′-Methyl-isoscutellarein 7-O-β-D-[6′″-O-acetyl]-allopyranosyl-(1→2)-β-D-glucopyranoside (**21**)	[79]
Section Eriostomum			
*S. alpina* L.	Aerial parts ^#^	8-Hydroxyflavone-allosylglucosides (not determined)	[75]
	Leaves ^#^	Hypolaetin 7-O-acetyl-allosyl-(1→2)-glucoside (not determined), Isoscutellarein-7-O-acetyl-allosyl-glucoside (not determined), Hypolaetin-4′-methyl- 7-O- acetyl-allosyl-glucoside (not determined)	[5]
*S. byzantina* K. Koch.	Aerial parts	Apigenin (**1**), Apigenin 7-O-β-glucoside (**2**), Apigenin 7-(6″-E-*p*-coumaroyl)-β-D-glucopyranoside (**6**)	[33]
	Aerial parts	Apigenin 7-(6″-E-*p*-coumaroyl)-β-D-glucopyranoside (**6**), Isoscutellarein 7-O-β-D-allopyranosyl-(1→2)-[6″-O-acetyl]-β-D-glucopyranoside (**16**), 4′-Methyl-isoscutellarein-7-O-β-D-allopyranosyl-(1→2)-[6″-O-acetyl]-β-D-glucopyranoside (**20**)	[80]
*S. cretica* subsp. *smyrnaea* Rech. f.	Aerial parts ^#^	Apigenin (**1**)	[81]
*S. germanica* L.	Aerial parts ^#^	Hypolaetin 7-allosyl-(1→2)-glucoside monoacetyl, Isoscutellarein 7-allosyl-(1→2)-glucoside monoacetyl, Hypolaetin 7-allosyl-(1→2)-glucoside diacetyl, Isoscutellarein-7-allosyl-(1→2)-glucoside diacetyl(not determined)	[75]
	Leaves ^#^	Apigenin 7-O-glucoside (**2**), Chrysoeriol 7-O-acetyl-allosyl-glucoside (not determined), 4′-Methyl-hypolaetin 7-O-acetyl-allosyl-(1→2)-glucoside (not determined), Apigenin 7-O-*p*-coumaroyl-glucoside (not determined)	[5]
*S. heraclea* All.	Aerial parts ^#^	8-Hydroxyflavone-allosylglucosides (not determined)	[75]
*S. lanata* Crantz. (*=S. germanica* L. subsp. *germanica*)	Aerial parts	Apigenin 7-O-β-D-glucopyranoside (**2**), Apigenin 7-(3″-Z-*p*-coumaroyl)-β-D-glucopyranoside (**5**), Apigenin 7-(6″-Z-*p*-coumaroyl)-β-D-glucopyranoside (**7**), Apigenin 7-O-(3′′,6′′-di-O-E-*p*-coumaroyl)-β-D-glucopyranoside (Anisofolin A) (**8**), Isoscutellarein 7-O-[6′″-O-acetyl]-β-D-allopyranosyl-(1→2)-β-D-glucopyranoside (**15**), Isoscutellarein 4′-methyl ether 7-O-β-D-[6′″-O-acetyl]-allopyranosyl-(1→2)-β-D-glucopyranoside (**21**), 4′-Methyl-hypolaetin-7-O-[6′″-O-acetyl]-β-D-allopyranosyl-(1→2)-β-D-glucopyranoside (**56**)	[82]
*S. spectabilis* Choisy ex DC.	Epigeal parts	Isostachyflaside (**25**), Spectabiflaside (**28**), Scutellarein 7-O-β-D-mannopyranosyl-(1→2)-β-D-glucopyranoside (stachyflaside) (**31**)	[83]
*S. thirkei* K. Koch.	Whole plant ^#^	Apigenin (**1**)	[84]
*S. tmolea* Boiss.	Aerial parts ^#^	Apigenin (**1**), Apigenin-7-O-glucoside (**2**)	[85]
*S. tymphaea* Hausskn. (=*S. germanica* subsp. *tymphaea* (Hausskn.) R. Bhattacharjee)	Flowering aerial parts	Isoscutellarein 7-O-[6′″-O-acetyl]-β-D-allopyranosyl-(1→2)-β-D-glucopyranoside (**15**), 4′-Methyl-isoscutellarein 7-O-β-D-[6′″-O-acetyl]-allopyranosyl-(1→2)-β-D-glucopyranoside (**21**), 4′-Methyl-hypolaetin-7-O-[6′″-O-acetyl]- allopyranosyl -(1→2)-[6″-O-acetyl]-glucopyranoside (**58**)	[86]
Section Fragilicaulis			
*S. subnuda* Montbret & Aucher ex Benth	Aerial parts	Ιsoscutellarein 7-O-allosyl-(1→2)-glucoside ^#^ (**13**), Isoscutellarein 7-O-[6″′-O-acetyl]-allosyl-(1→2)-glucoside (**15**), 4′-Methyl-isoscutellarein-7-O-β-D-allopyranosyl-(1→2)-β-D-glucoside ^#^ (**19**), 4′-Methyl-isoscutellarein 7-O-β-D-[6′″-O-acetyl]-allopyranosyl-(1→2)-β-D-glucoside (**21**), 4′-Methyl-isoscutellarein-7-O-[6′″-O-acetyl]-allosyl(1→2)-[6″-O-acetyl]-glucoside ^#^ (**24**)	[87]
Section Olisia			
*S. atherocalyx* C. Koch		Stachyflaside (**31**)	[72]
		Diacetylstachyflaside (not determined), Diacetylspectabiflaside (not determined), Spectabiflaside (**28**)	[88]
		5,8,4′-Trihydroxy-3′-methoxy-7-O-(β-D-glucopyranosyl-2″-O-β-D-mannopyranosyl)-flavone (Spectabiflaside) (**28**), Acetyl-sectabiflaside (not determined),	[89]
		Acetyl-isostachyflaside (**26**), Di-acetyl-isostachyflaside (**27**), Spectabiflaside (**28**)	[90]
	Leaves ^#^	Isoscutellarein 7-O-[6′″-O-acetyl]-β-D-allopyranosyl-(1→2)-β-D-glucopyranoside (**15**), 4′-Methyl-isoscutellarein-7-O-β-D-[6′″-O-acetyl]-allopyranosyl-(1→2)-β-D-glucopyranoside (**21**), 4′-Methyl-hypolaetin-7-O-[6′″-O-acetyl]-β-D-allopyranosyl-(1→2)-β-D-glucopyranoside (**56**)	[91]
*S. angustifolia* M. Bieb.		Isoscutellarein 7-O-[6′″-O-acetyl]-β-D-allopyranosyl-(1→2)-β-D-glucopyranoside (**15**), 4′-Methyl-isoscutellarein 7-O-β-D-[6′″-O-acetyl]-allopyranosyl-(1→2)-β-D-glucopyranoside (**21**)	[92]
*S. annua* (L.) L.	Epigeal parts	4′-Methyl-isoscutellarein (**12**), 7-O-β-D-glucopyranosyl- 5,6-dihydroxy-4′-methoxyflavone (Stachannin A) (**32**), 4′-Methoxy-scutellarein-7-[O-β-D-mannopyranosyl-(1→2)-β-D-glucopyranoside] (Stachannoside B) (**33**)	[93]
	Leaves ^#^	Isoscutellarein 7-O-[6′″-O-acetyl]-β-D-allopyranosyl-(1→2)-β-D-glucopyranoside (**15**), 4′-Methyl-isoscutellarein-7-O-β-D-[6′″-O-acetyl]-allopyranosyl-(1→2)-β-D-glucopyranoside (**21**), 4′-Methyl-hypolaetin-7-O-[6′″-O-acetyl]-β-D-allopyranosyl-(1→2)-β-D-glucopyranoside (**56**)	[92]
	Aerial parts	4′-Methyl-isoscutellarein 7-O-β-D-[6′″-O-acetyl]-allopyranosyl-(1→2)-β-D-glucopyranoside (**21**)	[94]
	Aerial parts	4′-O-Methyl-isoscutellarein-7-O-[4′″-O-acetyl]allopyranosyl-(1→2)- glucopyranoside (Annuoside) (**23**)	[95]
	Subterranean organs	4′-O-Methyl-isoscutellarein (**12**), 4′-O-Methyl-isoscutellarein 7-O-(6′″-O-acetyl)allopyranosyl-(1→2)-glucopyranoside (**21**)	[95]
*S.annua* (L.) L. subsp. *annua*	Flowering aerial parts	4′-Methyl-isoscutellarein 7-O-β-D-[6′″-O-acetyl]-allopyranosyl-(1→2)-β-D-glucopyranoside (**21**), Hypolaetin 7-O-[6′″-O-acetyl]-allosyl-(1→2)-[6″-O-acetyl]-glucopyranoside (**53**), 4′-Methyl-hypolaetin-7-O-[6′″-O-acetyl]-β-D-allopyranosyl-(1→2)-β-D-glucopyranoside (**56**)	[52]
*S. beckeana* Dörfler & Hayek	Leaves ^#^	Isoscutellarein 7-O-[6′″-O-acetyl]-β-D-allopyranosyl-(1→2)-β-D-glucopyranoside (**21**), 4′-Methyl-hypolaetin-7-O-[6′″-O acetyl]-β-D-allopyranosyl-(1→2)-β-D-glucopyranoside (**56**)	[92]
*S. bombycina* Boiss.	Aerial parts	Apigenin 7-(6″-E-*p*-coumaroyl)-β-D-glucopyranoside (**6**), Stachyspinoside (**44**)	[96]
*S. parolinii* Vis.	Leaves ^#^	Isoscutellarein 7-O-[6′″-O-acetyl]-β- D-allopyranosyl-(1→2)-β-D-glucopyranoside (**15**), 4′-Methyl-hypolaetin-7-O-[6′″-O-acetyl]-β-D-allopyranosyl-(1→2)-β-D-glucopyranoside (**56**)	[92]
*S. leucoglossa* Griseb.	Leaves ^#^	Isoscutellarein 7-O-[6′″-O-acetyl]-allosyl(1→2)-[6″-O-acetyl]-glucoside (**17**), 4′-Methyl-isoscutellarein 7-O-β-D-[6′″-O-acetyl]-allopyranosyl-(1→2)-β-D-glucopyranoside (**21**), 4′-Methyl-hypolaetin-7-O-[6′″-O-acetyl]-β-D-allopyranosyl-(1→2)-β-D-glucopyranoside (**56**)	[92]
*S. neglecta* Klok. ex Kossko (*=S. annua* (L.) L.)		Apigenin (**1**), Apigenin 7-O-β-D-glucoside (**2**), Luteolin (**34**), Luteolin 7-O-β-D-glucoside (**37**)	[97]
*S. recta* L.	Leaves	Isoscutellarein 7-O-[6′″-O-acetyl]-allosyl(1→2)-[6″-O-acetyl]-glucoside (**17**), 4′-Methyl-isoscutellarein 7-O-β-D-[6′″-O-acetyl]- allosyl](1→2)-β-D-glucoside (**21**), 4′-Methyl-hypolaetin 7-O-[6′″-O-acetyl]-β-D-allosyl(1→2)-β-D-glucoside (**56**)	[91,92]
	Aerial parts	Apigenin 7-(3″-E-p-coumaroyl)-β-D-glucopyranoside (**4**), Apigenin 7-(6″-E-*p*-coumaroyl)-β-D-glucopyranoside (**6**), Isoscutellarein 7-O-[allosyl(1→2)]- glucopyranoside (**13**), Isoscutellarein 7-O-[6′″-O-acetyl]-β-D-allosyl-(1→2)-β-D-glucopyranoside (**15**), Isoscutellarein 7-O-[6′″-O-acetyl]-allosyl(1→2)-[6″-O-acetyl]- glucopyranoside (**17**), 4′-Methylisoscutellarein 7- O-[allosyl-(1→2)]- glucopyranoside (**19**), 4′-Methyl-isoscutellarein 7-O-β-D-[6″-O-acetyl]-allopyranosyl-(1→2)-β-D-glucopyranoside (**20**), 4′-Methyl-isoscutellarein 7-O-β-D-[6′″-O-acetyl]- allosyl-(1→2)-β-D-glucopyranoside (**21**), 4′-Methyl-isoscutellarein 7-O-[6′″-O-acetyl]-allosyl-(1→2)-[6″-O-acetyl]-glucoside (**24**), Hypolaetin 7-O-allosyl-(1→2)-glucopyranoside ^#^ (**50**), 4′-Methyl-hypolaetin 7-O-allosyl(1→2)-glucoside ^#^ (**55**), 4′-Methyl-hypolaetin-7-O-[6″-O-acetyl]-allosyl-(1→2)- glucopyranoside (**57**), 4′-Methyl-hypolaetin 7-O-[6′″-O-acetyl]-allosyl-(1→2)-[6″-O-acetyl]- glucopyranoside (**58**)	[14]
*S. labiosa* Bertol. (=*S. recta* subsp. *labiosa* (Bertol.) Briq.)	Leaves	Isoscutellarein 7-O-β-D-[6′″-O-acetyl]-allopyranosyl-(1→2)-β-D-glucopyranoside (**15**), 4′-Methyl-isoscutellarein-7-O-β-D-[6′″-O-acetyl]-allopyranosyl-(1→2)-β-D-glucopyranoside (**21**), 4′-Methyl-hypolaetin-7-O-β-D-[6′″-O-acetyl]-allopyranosyl-(1→2)-β-D-glucopyranoside (**56**)	[92]
*S. subcrenata* Vis.(=*S. recta* L. subsp. *subcrenata* (Vis.) Briq.)	Leaves	Isoscutellarein 7-O-β-D-[6′″-O-acetyl]-allopyranosyl-(1→2)-β-D-glucopyranoside (**15**), 4′-Methyl-isoscutellarein-7-O-β-D-[6′″-O-acetyl]-allopyranosyl-(1→2)-β-D-glucopyranoside (**21**), 4′-Methyl-hypolaetin-7-O-β-D-[6′″-O-acetyl]-allopyranosyl-(1→2)-β-D-glucopyranoside (**56**)	[92]
*S. baldaccii* (Maly) Hand.—Mazz. (=*S. recta* L. subsp. *baldaccii* (K. Maly) Hayek)	Leaves ^#^	Isoscutellarein 7-O-β-D-[6′″-O-acetyl]-allopyranosyl]-(1→2)-β-D-glucopyranoside (**15**), 4′-Methyl-isoscutellarein-7-O-β-D-[6′″-O-acetyl]-allopyranosyl-(1→2)-β-D-glucopyranoside (**21**)	[92]
*S. spinosa* L.	Aerial parts	Chrysoeriol 7-O-[6′″-O-acetyl-allosyl]-(1→2)-glucoside (Stachyspinoside) (**44**)	[98]
	Aerial parts	Chrysoeriol 7-O-[6′′-O-acetyl-allosyl]-(1→2)-glucoside (Isostachyspinoside) (**45**)	[99]
*S. tetragona* Boiss. & Hayek	Leaves ^#^	Isoscutellarein 7-O-[6′″-O-acetyl]-β-D-allopyranosyl-(1→2)-β-D-glucopyranoside (**15**), 4′-Methyl-isoscutellarein 7-O-β-D-[6′″-O-acetyl]-allopyranosyl-(1→2)-β-D-glucopyranoside (**21**)	[92]
	Aerial parts	Isoscutellarein 7-O-[6′″-O-acetyl]-β-D-allopyranosyl-(1→2)-β-D-glucopyranoside (**15**), Isoscutellarein 7-O-[6′″-O-acetyl]- β-D-allosyl-(1→2)-[6″-O-acetyl]-β-D glucopyranoside (**17**)	[100]
Section Swainsoniana			
*S. anisochila* Vis. & Pancic	Leaves	Isoscutellarein 7-O-β-D-allopyranosyl-(1→2)-β-D-glucopyranoside (**13**), Isoscutellarein 7-O-[6′″-O-acetyl]-β-D-allopyranosyl-(1→2)-β-D-glucopyranoside (**15**), Isoscutellarein 7-O-[6′″-O-acetyl]- β-D-allosyl-(1→2)-[6″-O-acetyl]-β-D-glucopyranoside (**17**), 4′-Methyl-isoscutellarein-7-O-β-D-allopyranosyl-(1→2)-β-D-glucopyranoside (**19**), Hypolaetin 7-O-[6′″-O-acetyl]-β-D-allopyranosyl-(1→2)-β-D-glucopyranoside (**51**), Hypolaetin 7-O-[6′″-O-acetyl]- β-D-allopyranosyl-(1→2)-[6″-O-acetyl]-β-D glucopyranoside (**53**), 4′-Methyl-hypolaetin-7-O-[6′″-O-acetyl]-β-D-allopyranosyl-(1→2)-β-D-glucopyranoside (**56**), 4′-Methyl-hypolaetin 7-O-[6′″-O-acetyl]-β-D-allosyl-(1→2)-[6″-O-acetyl]-β-D glucopyranoside (**58**)	[101]
	Leaves	Apigenin 7-O-(*p*-coumaroyl)-β-D-glucopyranoside (not determined)	[5]
*S. decumbens* Pers. (=*S. mollissima* Willd.)	Aerial parts ^#^	8-Hydroxyflavone-allosylglucosides (not determined)	[75]
*S. menthifolia* Vis. (=*S. grandiflora* Host.)	Leaves ^#^	Isoscutellarein 7-O-β-D-[6′″-O-acetyl]-β-D-allopyranosyl-(1→2)-β-D-glucopyranoside (**15**), 4′-Methyl-isoscutellarein-7-O-β-D-[6′″-O-acetyl]-β-D allopyranosyl-(1→2)-β-D-glucopyranoside (**21**)	[92]
*S. swainsonii* Benth. subsp. *swainsonii*	Aerial parts	Apigenin (**1**), Apigenin 7-O-β-D-glucopyranoside (**2**), Apigenin 7-O-β-D-glucoside (**2**), Luteolin 7-O-β-D-glucopyranoside (**37**), Chrysoeriol (**42**), Chrysoeriol 7-O-β-D-glucopyranoside (**43**), Stachyspinoside (**44**)	[102]
*S. swainsonii* subsp. *argolica* (Boiss.) Phitos and Damboldt	Aerial parts	Apigenin (**1**), Luteolin 7-O-β-D-glucopyranoside (**37**), Chrysoeriol (**42**), Chrysoeriol-7-O-β-D-glucopyranoside (**43**), Chrysoeriol 7-(3″-E-*p*-coumaroyl)-β-D-glucopyranoside (**46**)	[102]
*S. swainsonii* subsp. *melangavica* D. Persson	Aerial parts	Apigenin (**1**), Apigenin 7-O-β-D- glucopyranoside (**2**), Luteolin 7-O-β-D-glucopyranoside (**37**), Chrysoeriol-7-O-β-D-glucopyranoside (**43**), Stachyspinoside (**44**)	[102]
*S. swainsonii* subsp. *scyronica* (Boiss.) Phitos and Damboldt	Aerial parts	Apigenin (**1**), Apigenin 7-O-β-D- glucopyranoside (**2**), Luteolin 7-O-β-D-glucopyranoside (**37**), Chrysoeriol-7-O-β-D-glucopyranoside (**43**), Stachyspinoside (**44**)	[102]
*S. ionica* Halácsy	Aerial parts	Apigenin (**1**), Apigenin 7-(6″-E-*p*-coumaroyl)-β-D-glucopyranoside (**6**), Isoscutellarein 7-O-[6′″-O-acetyl]-β-D-allopyranosyl-(1→2)-β-D-glucopyranoside (**15**), 4′-Methyl-isoscutellarein 7-O-β-D-[6′″-O-acetyl]-β-D-allopyranosyl-(1→2)-β-D-glucopyranoside (**21**)	[20]
Section Stachys			
*S. sieboldii* Miq. (=*S. affinis* Bunge)	Aerial parts	Isoscutellarein 7-O-[6′″-O-acetyl]-β-D-allosyl]-(1→2)-β-D-glucoside (**15**), 4′-Methyl-isoscutellarein 7-O-[6′″-O-acetyl]-β-D-allopyranosyl-(1→2)-β-D-glucoside (**21**)	[20]
*S. mialhesii* Noé	Aerial parts	Apigenin 7-(6″-E-*p*-coumaroyl)-β-D-glucopyranoside (**6**), Isoscutellarein 7-O-[6′″-O-acetyl]-β-D-allopyranosyl-(1→2)-β-D-glucopyranoside (**15**)	[103]
*S. palustris* L.		5-(glycuroglucosyl)-7-methoxybaicalein (Palustrin) (**63**), 5-(glucuronosyl)-7-methoxybaicalein (Palustrinoside) (**64**)	[104]
	Leaves ^#^	Vicenin-2 (**10**), Apigenin 7-O-*p*-coumaroyl-β-D-glucopyranoside (not determined)	[5]
*S. sylvatica* L.	Aerial parts ^#^	8-Hydroxyflavone-allosyl-glucosides (not determined)	[75]
	Leaves ^#^	Chrysoeriol 7-O-acetylallosylglucoside (not determined), Apigenin 7-O-*p*-coumaroyl-β-D-glucopyranoside (not determined)	[5]
*S. plumosa* Griseb.	Leaves ^#^	Apigenin-7-O-β-D-glucoside (**2**), Luteolin 7-O-β-D-glucoside (**37**), Chrysoeriol 7-O-acetyl-allosyl-glucoside (not determined), Isoscutellarein 7-O-acetyl-allosyl-glucoside (not determined), Apigenin 7-O-*p*-coumaroyl-β-D-glucopyranoside (not determined)	[5]
Section Zietenia			
*S. lavandulifolia* Vahl.	Aerial parts	Apigenin (**1**), Hydroxygenkwanin (Luteolin 7-Methyl ether) (**35**), Chrysoeriol (**42**)	[13]
*S. tibetica* Vatke	Roots	Apigenin 7-O-β-D-glucoside (**2**)	[66]
**Subgenus *Betonica***			
Section Betonica			
*S. alopecuros* (L.) Benth.	Aerial parts	*p*-coumaroyl-glucosides (not determined) ^#^	[75]
	Leaves ^#^	Isoscutellarein 7-O-glucoside (**11a**), Luteolin 7-O-glucuronide (**36**), Luteolin 7-O-glucoside (**37**), Chrysoeriol 7-O-glucoside (**43**), Hypolaetin 7-O-glucoside (**49**), Hypolaetin 7-O-glucuronide (**49a**), Selgin 7-O-glucoside (**59**), Tricin 7-O-glucuronide (**60**), Tricin 7-O-glucoside (**61**), Apigenin 7-O-*p*-coumaroyl glucopyranoside (not determined)	[5]
*S. foliosa* Regel. (=*S*. *betoniciflora* Rupr.; *Betonica foliosa* Rupr.)		Four flavonoids (not determined)	[105]
*S. monieri* (Gouan) P.W. Ball. (=*S. officinalis* (L.) Trevis subsp. *officinalis*)	Aerial parts	*p*-coumaroyl-glucosides (not determined) ^#^	[75]
*S. officinalis* (L.) Trevis (=*Betonica officinalis* L.)		Apigenin (**1**), 5, 6, 4′-trihydroxyflavone-7-O-β-D-glucoside (**30**)	[20]
	Leaves ^#^	Apigenin 8-C-glucoside (Vitexin) (**9**), Luteolin 7-O-glucuronide (**36**), Luteolin 6-C-glucoside (isoorientin) (**38**), Tricin 7-O-glucuronide (**60**), Tricin 7-O-glucoside (**61**), Tricetin 3′,4′,5′-trimethyl-7-O-glucoside (**62**), Apigenin 7-O-*p*-coumaroyl glucopyranoside (not determined)	[5]
	Aerial parts	*p*-coumaroyl-glucosides (not determined) ^#^	[75]
Section Macrostachya			
*S. scardica* Griseb. (=*Betonica scardica* Griseb.)	Leaves ^#^	Apigenin 8-C-glucoside (**9**), Luteolin 7-O-glucoside (**37**), Luteolin 6-C-glucoside (**38**), Hypolaetin 7-O-glucoside (**49**), Selgin 7-O-glucoside (**59**), Tricin 7-O-glucuronide (**60**), Tricin 7-O-glucoside (**61**), Tricetin 3′,4′,5′-trimethyl-7-O-glucoside (isolation) (**62**), Apigenin 7-O-*p*-coumaroyl glucopyranoside (not determined)	[5]

^#^ identified compounds by means of HPLC, LC-MS, etc.

**Table 3:** Poly-methylated flavonoids from *Stachys* spp.

Species	Plant Parts	Compound	Ref	
Subgenus *Stachys*				
Section Ambleia				
*S. aegyptiaca* Pers.	Aerial parts	Xanthomicrol (**69**), Sideritiflavone (**70**), 5-Hydroxy-6,7,8,3′,4′-pentamethoxyflavone (**75**), 5,4′-Dihydroxy - 6,7,8,3′-tetramethoxyflavone (**76**), 5,3′,4′-Trihydroxy-3,6,7,8-tetramethoxyflavone (**82**), Calycopterin (**83**), Chrysosplenetin (**84**), 5-Hydroxy-3,6,7,8,4′- pentamethoxyflavone (**88**), 5,4′-Dihydroxy -3,6,7,8,3′- pentamethoxyflavone (**89**)	[68]	
	Aerial parts	5,7,3′-Trihydroxy-6,4′-dimethoxyflavone (**67**), 5,7,3′-Trihydroxy-6,8,4′-trimethoxyflavone (**68**)	[70]	
	Aerial parts	Xanthomicrol (**69**), Eupatilin-7-methyl ether (**73**), Calycopterin (**83**), 5-Hydroxy-3,6,7,4′-tetramethoxy flavone (**85**), 5,8-Dihydroxy-3,6,7,4′-tetramethoxy flavone (**86**), 5-Hydroxy-auranetin (**88**), 4′-Hydroxy-3,5,7,3′- tetramethoxy flavone (**90**)	[106]	
*S. schtschegleevii* Sosn. ex Grossh.	Stems	Cirsimaritin (**66**), Xanthomicrol (**69**)	[74]	
Section Aucheriana				
*S. glutinosa* L.		Xanthomicrol (**69**), Sideritiflavone (**70**), 8-Methoxycirsilineol (**71**), Eupatilin (**72a**)	[107]	
Section Candida				
*S. candida* Bory & Chaubard	Aerial parts	Xanthomicrol (**69**), Calycopterin (**83**)	[77,78]	
*S. chrysantha* Boiss. and Heldr.	Aerial parts	Xanthomicrol (**69**), Calycopterin (**83**)	[77]	
Section Swainsoniana				
*S. swainsonii* Benth. subsp. *swainsonii*	Aerial parts	Eupatorin (**72**), Penduletin (**81**), 5-Hydroxyauranetin (**88**)	[102]	
*S. swainsonii* subsp. *argolica* (Boiss.) Phitos and Damboldt	Aerial parts	Xanthomicrol (**69**), Eupatorin (**72**), Salvigenin (**74**)	[102]	
*S. swainsonii* subsp. *melangavica* D. Persson	Aerial parts	Eupatorin (**72**), 5-Hydroxyauranetin (**88**)	[102]	
*S. swainsonii* subsp. *scyronica* (Boiss.) Phitos and Damboldt	Aerial parts	Eupatorin (**72**), Penduletin (**81**), 5-Hydroxyauranetin (**88**)	[102]	
*S. ionica* Halácsy	Aerial parts	Xanthomicrol (**69**), Salvigenin (**74**), Chrysosplenetin (**84**), 5-Hydroxy-3,6,7,4′-tetramethoxyflavone (**85**), Casticin (**87**)	[20]	
*S. lavandulifolia* Vahl.	Aerial parts	Velutin (Luteolin 7,3′-dimethyl ether) (**65**), Viscosine (5,7,4′-trihydroxy-3,6-dimethoxyflavone (**78**), Kumatakenin (Kaempferol 3,7-dimethyl ether) (**79**), Pachypodol (Quercetin 3,7,3′-trimethyl ether) (**80**), Penduletin (**81**), Chrysosplenetin (**84**),	[13]	
**Subgenus *Betonica***				
Section Betonica				
*S. officinalis* (L.) Trevis = (*Betonica officinalis* L.)		5,4′-Dyhydroxy-7,3′,5′-trimethoxyflavone (**77**)	[20]	

**Table 4:** Flavonols from *Stachys* spp.

Species	Plant Parts	Compound	Ref
Subgenus *Stachys*			
Section Eriostomum			
*S. cretica* subsp. *smyrnaea* Rech. f.	Aerial parts ^#^	Kaempferol (**91**)	[81]
Section Olisia			
*S. tetragona* Boiss. & Hayek	Aerial parts	Kaempferol (**91**)	[100]
Section Swainsoniana			
*S. swainsonii* Benth. subsp. *swainsonii*	Aerial parts	Isorhamnetin (**92**)	[99]
*S. swainsonii* subsp. *argolica* (Boiss.) Phitos and Damboldt	Aerial parts	Isorhamnetin (**92**)	[99]
Section Stachys			
*S. palustris* L.	Leaves ^#^	Quercetin-3-O-rutinoside (**93**), Isorhamnetin-3-O-rutinoside (**94**)	[5]

^#^ identified compounds by means of HPLC, LC-MS, etc.

**Table 5:** Flavanones from *Stachys* spp.

Species	Plant Parts	Compound	Ref
Subgenus *Stachys*			
Section Ambleia			
*S. aegyptiaca* Pers.	Aerial parts	Naringenin (**96**)	[69]
Section Eriostomum			
*S. cretica* subsp. *smyrnaea* Rech. f.	Aerial parts ^#^	Hesperidin (**97**)	[81]
Section Swainsoniana			
*S. swainsonii* Benth. subsp. *swainsonii*	Aerial parts	Eriodictyol (**95**)	[102]
*S. swainsonii* subsp. *argolica* (Boiss.) Phitos and Damboldt	Aerial parts	Eriodictyol (**95**)	[102]
*S. swainsonii* subsp. *melangavica* D. Persson	Aerial parts	Eriodictyol (**95**)	[102]
*S. swainsonii* subsp. *scyronica* (Boiss.) Phitos and Damboldt	Aerial parts	Eriodictyol (**95**)	[102]

**Table 6:** Biflavonoid from *Stachys* spp.

Species	Plant Parts	Compound	Ref
Subgenus *Stachys*			
Section Ambleia			
*S. aegyptiaca* Pers.	Aerial Parts	Diapigenin-7-O-(6″-trans,6″-cis-*p*, *p*′-dihydroxy-µ-truxinyl)glucoside (stachysetin) (**98**)	[69]
Section Eriostomum			
*S. lanata* Crantz. (*=S. germanica* L. subsp. *germanica*)	Aerial parts	Stachysetin (**98**)	[82]
Section Candida			
*S. iva* Griseb.	Flowering aerial parts	Stachysetin (**98**)	[56]

**Table 7:** Phenolic derivatives from *Stachys* spp.

Species	Plant Parts	Compound	Ref
Subgenus *Stachys*			
Section Candida			
*S. candida* Bory & Chaubard	Aerial parts	Chlorogenic acid (**103**)	[78]
*S. iva* Griseb	Flowering aerial parts	Chlorogenic acid (**103**)	[56]
Section Eriostomum			
*S. cretica* subsp. *smyrnaea* Rech. f.	Aerial parts ^#^	Chlorogenic acid (**103**)	[81]
*S. cretica* subsp. *vacillans* Rech. f.	Aerial parts ^#^	Vanillic acid (**100**), Syringic acid (**101**), Chlorogenic acid (**103**)	[105]
*S. cretica* subsp. *mersinaea* (Boiss.) Rech. f.	Aerial parts ^#^	Chlorogenic acid (**103**)	[108]
*S. lanata* Crantz. (=*S. germanica* L. subsp. *germanica*)	Roots	Chlorogenic acid (**103**)	[82]
*S. tmolea* Boiss	Aerial parts ^#^	4-Hydroxybenzoic acid (**99**), Chlorogenic acid (**103**)	[85]
*S. thirkei* K. Koch	Aerial parts ^#^	Chlorogenic acid (**103**)	[84]
*S. germanica* L. subsp. *salviifolia* (Ten.) Gams.	Aerial parts	Arbutin (**107**)	[109]
Section Olisia			
*S. atherocalyx* C. Koch.		Νeochlorogenic acid (**105**), *p*-Coumaric acid (**106**), Caffeic acid (**108**)	[110]
*S. recta* L.	Aerial parts ^#^	1-Caffeoylquinic acid (**102**), Chlorogenic acid (**103**), 4-Caffeoylquinic acid (**104**)	[14]
Section Stachys			
*S. palustris* L.		1-Caffeoylquinic acid (**102**), Chlorogenic acid (**103**), 4-Caffeoylquinic acid (**104**), Caffeic acid (**108**)	[104]
		Cryptochlorogenic acid (**104**), Neochlorogenic acid (**105**)	[23]
**Subgenus *Betonica***			
Section Betonica			
*S. officinalis* L. (=*Betonica officinalis* L.)	Leaves ^#^	Chlorogenic acid (**103**)	[111]

^#^ identified compounds by means of HPLC, LC-MS, etc.

**Table 8:** Acetophenone glycosides from *Stachys* spp.

Species	Plant Parts	Compound	Ref
Subgenus *Stachys*			
Section Eriostomum			
*S. lanata* Crantz. (=*S. germanica* L. subsp. *germanica*)	Roots	Androsin (**109**), Neolloydosin (**110**), Glucoacetosyringone (**111**)	[82]

**Table 9:** Lignans from *Stachys* spp.

Species	Plant Parts	Compound	Ref
Subgenus *Stachys*			
Section Stachys			
*S. mialhesii* Noé	Aerial Parts	(+) -Sesamin (**112**), (+) -Paulownin (**113**)	[103]
Section Olisia			
*S. tetragona* Boiss. & Heldr.	Aerial parts	(7S-8R)-Urolignoside (**114**)	[100]

**Table 10:** Phenylethanoid glycosides from *Stachys* spp.

Species	Plant Parts	Compound	Ref
Subgenus *Stachys*			
Section Ambleia			
*S. schtschegleevii* Sosn. ex Grossh.	Stems	Acteoside (**118**), Betunyoside F (**128**)	[74]
Section Candida			
*S. candida* Bory & Chaubard	Aerial parts	Acteoside (**118**)	[78]
*S. iva* Griseb.	Flowering aerial parts	Acteoside (**118**), Leucosceptoside A (**131**), Lavandulifolioside (**129**)	[56]
Section Eriostomum			
*S. byzantina* Κ. Koch	Aerial parts	Verbascoside (**118**), 2′-O-Arabinosyl verbascoside (**122**), Aeschynanthoside C (**133**)	[33]
*S. cretica* L. subsp. *vacillans* Rech. f.	Aerial parts ^#^	Verbascoside (**118**)	[112]
*S. germanica* L. subsp. *salviifolia* (Zen.) Gams	Aerial parts	Verbascoside (**118**)	[109]
*S. lanata* Crantz (=*S. germanica* L. subsp. *germanica*)	Aerial parts	Leonoside B (**134**), Martynoside (**135**)	[82]
	Roots	Rhodioloside (**115**), Verbasoside (**116**), Verbascoside (**118**), Isoacteoside (**119**), Darendoside B (**120**), Campneoside II (**121**), 2-Phenylethyl-D-xylopyranosyl-(1→6)-D-glucopyranoside (**117**), Campneoside I (**136**)	[82]
*S. tymphaea* Hausskn. (=*S. germanica* subsp. *tymphaea* (Hausskn.) R. Bhattacharjee)	Flowering aerial parts	Verbascoside (**118**), Stachysoside A (**129**)	[86]
Section Olisia			
*S. recta* L.	Aerial parts	Acteoside (**118**), Isoacteoside (**119**), β-OH-Acteoside (**121**), Betunyoside E (**127**), Campneoside I (**136**), Forsythoside B (**137**), β-OH-Forsythoside B methyl ether (**138**)	[14]
*S. tetragona* Boiss. & Heldr.	Aerial parts	Acteoside (**118**), Betonioside F (**128**), Leucosceptoside A (**131**), Stachysoside D (**134**), Forsythoside B (**137**), Lamiophloside A (**141**)	[100]
Section Stachys			
*S. affinis* Bunge (=*S. sieboldii* Miq.)	Tubers	Acteoside (**118**), Leucosceptoside A (**131**), Martynoside (**135**)	[27]
		Stachysosides A (**129**), B (**139**), C (**140**)	[113]
*S. riederi* Cham.	Whole plants	Acteoside (**118**), Campneoside II (**121**), Lavandulifolioside (**129**), Leonoside A (**139**)	[114]
Section Zietenia			
*S. lavandulifolia* Vahl	Aerial parts	Acteoside (**118**), Lavandulifolioside (**129**)	[115]
	Aerial parts	Verbascoside (**118**), Lavandulofolioside A (**129**), Lavandufolioside B (**130**), Leucosceptoside A (**131**)	[12]
	Aerial parts	Acteoside (**118**)	[116]
**Subgenus *Betonica***			
Section Betonica			
*S. macrantha* (C. Koch.) Stearn (=*Betonica grandiflora* Willd.)	Aerial parts	Verbascoside (**118**), Leucosceptoside A (**131**), Martynoside (**135**), Lavandulifolioside (**129**)	[117]
*S. officinalis* (L.) Trevis. (=*Betonica officinalis* L.)	Aerial parts	Acteoside (**118**), Acteoside isomer (isoacteoside) (**119**), Campneoside II (**121**), Betonyosides A-F (**123–128**), Leucosceptoside B (**132**), Forsythoside B (**137**)	[118]
*S. alopecuros* (L.) Benth subsp. *divulsa* (Ten.) Grande	Flowering aerial parts	Verbascoside (**118**)	[119]
**Former *Stachys* species**			
*S. parviflora* Benth. (=*Phlomidoschema parviflorum* (Benth.) Vved.)	Whole plant	Parvifloroside A (**142**), Parvifloroside B (**143**)	[120]

^#^ identified compounds by means of HPLC, LC-MS, etc.

**Table 11:** Phenylpropanoid glucosides from *Stachys* spp.

Species	Plant Parts	Compound	Ref
Subgenus *Stachys*			
Section Eriostomum			
*S. lanata* Crantz. (=*S. germanica* L. subsp. *germanica*)	Roots	Coniferin (**144**), Syringin (**145**)	[82]

**Table 12:** Iridoids from *Stachys* spp.

Species	Plant Parts	Compound	Ref
Subgenus *Stachys*			
Section Ambleia			
*S. inflata* Benth.		Ajugol (**146**), Ajugoside (**147**),	[121]
Section Aucheriana			
*S. glutinosa* L.	Aerial parts	Harpagide (**148**), Acetylharpagide (**150**), Monomelittoside (**165**), Melittoside (**166**), Allobetonicoside (**161**), 5-Allosyloxy-aucubin (**167**)	[122]
Section Campanistrum			
*S. ocymastrum* (L.) Briq. (=*S. hirta* L.)	Leaves	6β-Acetoxyipolamiide (**172**), 6β-Hydroxyipolamiide (**173**), Ipolamiide (**174**), Ipolamiidoside (**175**), Lamiide (**176**)	[123]
Section Candida			
*S. iva* Griseb.	Flowering Aerial parts	Harpagide (**148**), 8-Acetylharpagide (**150**), 8-*Epi-*loganic acid (**157**), Gardoside (**160**), 8-*Epi*-loganin (**159**), Monomelittoside (**165**), Melittoside (**166**)	[56]
Section Corsica			
*S. corsica* Pers.		Harpagide (**148**), Acetylharpagide (**150**)	[79]
Section Eriostomum			
*S. alpina* L.	Stems, Leaves ^#^	Ajugoside (**147**), Harpagide (**148**), Acetylharpagide (**150**), Harpagoside (**154**), Aucubin (**164**), Catalpol (**163**)	[124]
*S. balansae* Boiss. & Kotschy		Ajugol (**146**), Ajugoside (**147**)	[125]
*S. germanica* L.		Harpagide (**148**)	[125]
	Leaf, Inflorescence ^#^	Ajugoside (**147**), Harpagide (**148**), Acetylharpagide (**150**), Harpagoside (**154**), Aucubin (**164**), Catalpol (**163**)	[124]
*S. spectabilis* Choisy ex DC.		Ajugol (**146**), Ajugoside (**147**), Harpagide (**148**)	[125]
*S. byzantina* Κ. Koch.	Aerial parts ^#^	Ajugoside (**147**), Harpagide (**148**), Acetylharpagide (**150**), Harpagoside (**154**), Catalpol (**163**), Aucubin (**164**)	[124]
*S. germanica* L. subsp. *salviifolia* (Zen.) Gams	Flowering Aerial parts	Harpagide (**148**)	[86]
	Aerial parts	Ajugol (**146**), Harpagide (**148**), 7-Hydroxyharpagide (**149**), 5-Allosyloxy-aucubin (**167**)	[109]
*S. lanata* Crantz. (=*S. germanica* L. subsp. *germanica*)	Roots	Stachysosides E (**168**), G (**170**), H (**171**)	[82]
	Aerial parts	Stachysosides E (**168**), F (**169**)	[82]
*S. tymphaea* Hausskn. (=*S. germanica* subsp. *tymphaea* (Hausskn.) R. Bhattacharjee)	Aerial parts	Harpagide (**148**)	[86]
Section Olisia			
*S. angustifolia* M. Bieb.		Ajugoside (**147**), Acetylharpagide (**150**), Harpagide (**148**), Melittoside (**166**)	[92]
*S. annua* (L.) L.		Ajugoside (**147**), Acetylharpagide (**150**), Melittoside (**166**)	[92]
*S. atherocalyx* C. Koch.		Ajugol (**146**), Harpagide (**148**), Acetylharpagide (**150**), Melittoside (**166**)	[92,125]
*S. beckeana* Dörfl. & Hayek		Harpagide (**148**), Ajugol (**146**), Acetylharpagide (**150**), Melittoside (**166**)	[92]
*S. iberica* M. Bieb.		Ajugol (**146**), Ajugoside (**147**), Harpagide (**148**), Acetylharpagide (**150**)	[121]
*S. recta* L.		Ajugol (**146**), Harpagide (**148**), Acetylharpagide (**150**), Melittoside (**166**)	[92]
	Leaves	8-Acetylharpagide (**150**), Melittoside^#^ (**166**)	[14]
	Aerial parts ^#^	Ajugoside (**147**), Harpagide (**148**), Acetylharpagide (**150**), Harpagoside (**154**), Catalpol (**163**), Aucubin (**164**)	[124]
*S. baldaccii* (Maly) Hand-Mazz (=*S. recta* L. subsp. *baldaccii* (K. Maly) Hayek)		Ajugol (**146**), Ajugoside (**147**), Harpagide (**148**), Acetylharpagide (**150**), Melittoside (**166**)	[92]
*S. subcrenata* Vis. (=*S. recta* subsp. *subcrenata*)		Ajugol (**146**), Harpagide (**148**), Acetylharpagide (**150**), Melittoside (**166**)	[92]
*S. labiosa* Bertol.		Ajugol (**146**), Harpagide (**148**), Acetylharpagide (**150**), Melittoside (**166**)	[92]
*S. leucoglossa* Griseb.		Ajugol (**146**), Harpagide (**148**), Acetylharpagide (**150**), Melittoside (**166**)	[92]
*S. spinosa* L.	Aerial parts	Ajugol (**146**), Harpagide (**148**), 7-O-Acetyl-8-*epi*-loganic acid (**158**)	[98]
*S. tetragona* Boiss. & Heldr.		Ajugol (**146**), Ajugoside (**147**), Harpagide (**148**), Acetylharpagide (**150**), Melittoside (**166**)	[92]
	Aerial parts	8-Acetyl-harpagide (**150**), 5-O-Allopyranosyl-monomelittoside (**167**)	[100]
Section Stachys			
*S. affinis* Bunge (= *S. sieboldii* Miq.)	Tubers	Harpagide (**148**), Acetylharpagide (**150**), Melittoside (**166**), 5-Allosyloxy-aucubin (**167**)	[27]
*S. palustris* L.	Aerial parts ^#^	Ajugoside (**147**), Harpagide (**148**), Acetylharpagide (**150**), Harpagoside (**154**), Catalpol (**163**), Aucubin (**164**)	[124]
*S. sylvatica* L.	Aerial parts ^#^	Ajugoside (**147**), Harpagide (**148**), Acetylharpagide (**150**), Harpagoside (**154**), Catalpol (**163**), Aucubin (**164**)	[124]
Section Swainsoniana			
*S. anisochila* Vis. & Pancic		Acetylharpagide (**150**), Melittoside (**166**)	[92]
*S. ionica* Halácsy		8-*epi-*loganic acid (**157**), Gardoside (**160**)	[20]
*S. menthifolia* Vis. (= *S. grandiflora* Host.)		Ajugol (**146**), Harpagide (**148**), Acetylharpagide (**150**), Melittoside (**166**)	[92]
	Aerial parts ^#^	Ajugoside (**147**) Harpagide (**148**), Acetylharpagide (**150**), Harpagoside (**154**), Catalpol (**163**), Aucubin (**164**)	[124]
Section Zietenia			
*S. lavandulifolia* Vahl.		Ajugol (**146**), Ajugoside (**147**)	[125]
	Aerial parts	Melittoside (**166**), Monomelittoside (**165**), 5-O-Allopyranosyl-monomelittoside (**167**)	[12]
**Subgenus** ***Betonica***			
Section Betonica			
*S. alopecuros* (L.) Benth subsp. *divulsa* (Ten.) Grande	Flowering aerial parts	Harpagide (**148**), Acetylharpagide (**150**), 4′-O-β-D-galactopyranosyl-teuhircoside (**162**)	[119]
*S. foliosa* Rupr. (=*S. betoniciflora* Rupr.; *Betonica foliosa* Rupr.)		Harpagide (**148**), Acetylharpagide (**150**)	[126]
*S. betonicaeflora* Rupr.		Harpagide (**148**), Acetylharpagide (**150**)	[126]
*S. macrantha* (C. Koch.) Stearn (=*Betonica grandiflora* Steph. ex Willd.)	Aerial parts	Ajugol (**146**), Ajugoside (**147**), Harpagide (**148**), 8-O-Acetyl-harpagide (**150**), Reptoside (**153**), Macranthoside [=8-O- (3, 4-dimethoxy-cinnamoyl-harpagide)] (**156**), Allobetonicoside (**161**)	[117]
*S. officinalis* (L.) Trevis. (*=Betonica officinalis* L.)	Aerial parts	Acetylharpagide (**150**), Reptoside (**153**), 6-O-Acetylmioporoside (**155**), Allobetonicoside (**161**)	[127]
		Harpagide (**148**), Acetylharpagide (**150**)	[128]
	Aerial parts ^#^	Ajugoside (**147**), Harpagide (**148**), Acetylharpagide (**150**), Harpagoside (**154**), Catalpol (**163**), Aucubin (**164**)	[124]
**Unknown Section**			
*S. grandidentata* Lindl. **	Aerial parts	Ajugol (**146**), Harpagide (**148**), Acetylharpagide (**150**), 5-Desoxy-harpagide (**151**), 5-Desoxy-8-acetyl-harpagide (**152**), Monomelittoside (**165**), Melittoside (**166**)	[129]

^#^ identified compounds#identified compounds by means of HPLC, LC-MS, etc; ** endemic species of Chile.

**Table 13:** Diterpenes from *Stachys* spp.

Species	Plant Parts	Compound	Ref
Subgenus *Stachys*			
section Ambleia			
*S. aegyptiaca* Pers.		Stachysolone (**177**), 11a,18-Dihydroxy-*ent*-kaur-16-ene (**210**)	[130]
	Aerial parts	Stachysperoxide (**189**), Stachysolone (**177**), 7,13-Diacetyl-stachysolone (**180**)	[131]
	Aerial parts	Stachaegyptin A-C (**190–192**), Roseostachenone (**184**), Stachysolone (**177**), 7,13-Diacetyl-stachysolone (**180**)	[106]
	Aerial parts	Stachaegyptins D, E (**193**, **194**)	[132]
	Aerial parts	Stachaegyptins A (**190**), F-H (**195–197**), Stachysperoxide (**189**)	[133]
*S. inflata* Benth.		Annuanone (**181**), Stachylone (**182**), Stachone (**183**)	[134]
Section Aucheriana			
*S. glutinosa* L.	Aerial parts	Roseostachenone (**184**), 3α,4α-Epoxyroseostachenol (**188**)	[107]
Section Eriostomum			
*S. balansae* Boiss. & Kotschy		Annuanone (**181**), Stachylone (**182**)	[134]
*S. lanata* Crantz. (=*S. germanica* L. subsp. *germanica*)		*Ent*-3α-acetoxy-kaur-16-en-19-oic acid (**207**), *Ent*-3α,19-dihydroxy-kaur-16-ene (**208**), *Ent*-3α-hydroxy-kaur-16-en-19-oic acid (**209**)	[135]
Section Mucronata			
*S. mucronata* Sieb.	Aerial parts	Ribenone [=3β-hydroxy-13-*epi*-*ent*-manoyl oxide] (**198**), Ribenol [=3-keto-13-*epi*-*ent*-manoyl oxide] (**199**)	[57]
Section Olisia			
*S. annua* (L.) L.		Stachysolone (**177**)	[136,137]
		Annuanone (**181**), Stachylone (**182**), Stachone (**183**)	[138]
*S. atherocalyx* C. Koch.		Annuanone (**181**), Stachylone (**182**), Stachone (**183**)	[134]
*S. distans* Benth	Aerial parts	(+)-6-Deoxyandalusol (**201**)	[139]
*S. iberica* M. Bieb.		Annuanone (**181**), Stachylone (**182**), Stachone (**183**)	[134]
*S. recta* L.	Aerial parts	7,13-Diacetate stachysolone (**180**), 7-Acetate stachysolone (**178**), 13-Acetate stachysolone (**179**)	[140]
Section Roseostachys			
*S. rosea* Boiss.	Aerial parts	Roseostachenone (**184**), Roseostachone (**185**), 13-*epi*-sclareol (**200**), Roseostachenol (**186**), Roseotetrol (**187**)	[141]
Section Stachys			
*S. mialhesii* Noé	Aerial parts	Horminone (**211**)	[103]
*S. palustris* L.		Annuanone (**181**)	[134]
*S. sylvatica* L.		Stachysic acid (**204**)	[142]
		Annuanone (**181**), Stachylone (**182**), Stachone (**183**)	[134]
		Stachysic acid (**204**), 6β-Hydroxy-*ent*-kaur-16-ene (**205**), 6β,18-Dihydroxy-*ent*-kaur-16-ene (**206**)	[142]
		Betolide (**214**)	[143]
Section Swainsoniana			
*S. ionica* Halácsy	Aerial parts	(+)-6-Deoxyandalusol (**201**)	[139]
*S. plumosa* Griseb.	Aerial parts	(+)-6-Deoxyandalusol (**201**), 13-*Epi*-jabugodiol (**202**), (+)-Plumosol (**203**)	[144]
Section Zietenia			
*S. lavandulifolia* Vahl.	Aerial parts	Stachysolone (**177**)	[116]
**Subgenus *Betonica***			
Section Betonica			
*S. officinalis* (L.) Trevis. (*=Betonica officinalis* L.)		Betolide (**214**)	[145]
		Betonicolide (**215**), Betonicosides A-D (**216–219**)	[145]
	Roots	Betolide (**214**)	[143]
*S. scardica* (Griseb.) Hayek (*=Betonica scardica* Griseb.)	Roots	Betolide (**214**)	[143]
**Former *Stachys* species**			
*S. parviflora* Benth. (=*Phlomidoschema parviflorum* (Benth.) Vved.)	Whole plant	Stachyrosane 1 (**212**) Stachyrosane 2 (**213**)	[133]

**Table 14:** Triterpene derivatives, Phytosterols and Phytoecdysteroids from *Stachys* spp.

Species	Plant Parts	Compound	Ref
Subgenus *Stachys*			
Section Eriostomum			
*S. byzantina* K. Koch	Aerial parts	Stigmasterol (**220**),	[17]
		β-Sitosterol (**221**), Lawsaritol (**223**), Stigmastan-3,5-dien-7-one (**224**)	[35]
*S. hissarica* Regel	-	20-Hydroxyecdysone (**239**), Polipodin B (**240**), Integristeron A (**241**), 2-Desoxy-20-hydroxyecdysone (**242**), 2-Desoxyecdyson (**243**)	[67]
Section Olisia			
*S. annua* (L.) L.	Aerial parts	β-Sitosterol (**221**), Ursolic acid (**226**)	[95]
*S. spinosa* L.	Aerial parts	Stigmasterol (**220**), β-Sitosterol (**221**), Oleanolic acid (**227**), 12α-Hydroxy-oleanolic lactone (**228**)	[99]
*S. tetragona* Boiss. & Heldr.	Aerial parts	Stigmasterol (**220**), β-Sitosterol (**221**), Oleanolic acid (**227**),	[100]
Section Stachys			
*S. palustris* L.		β-Sitosterol (**221**), α-amyrin (**225**)	[146]
*S. riederi* Cham.	Whole plant	Stachyssaponins I-VIII (**231–238**)	[147]
**Subgenus *Betonica***			
Section Betonica			
*S. alopecuros* (L.) Benth subsp. *divulsa* (Ten.) Grande	Flowering aerial parts	3-O-β-Sitosterol-glucoside (**222**)	[119]
**Former *Stachys* species**			
*S. parviflora* Benth. (=*Phlomidoschema parviflorum* (Benth.) Vved.)	Aerial parts	Stachyssaponin A (**229**), Stachyssaponin B (**230**)	[63]

**Table 15:** Megastigmane derivatives from *Stachys* spp.

Species	Plant Parts	Compound	Ref
Subgenus *Stachys*			
Section Eriostomum			
*S. byzantina* K. Koch.	Aerial parts	Byzantionoside A (**244**), Byzantionoside B (**245**), Icariside B2 (**246**), (6R, 9R)- and (6R, 9S)-3-oxo-α-ionol glucosides (**247**), Blumeol C glucoside (**248**)	[148]
*S. lanata* Crantz (=*S. germanica* L. subsp. *germanica*)	Aerial parts	Vomifoliol (**249**), Dehydrovomifoliol (**250**)	[82]
	Roots	Citroside A (**251**)	[82]

**Table 16:**
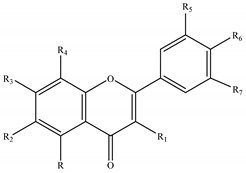
Chemical structures of flavones isolated from *Stachys* spp.

Name	R_1_	R_2_	R_3_	R_4_	R_5_	R_6_	R_7_
R=OH							
Apigenin (**1**)	H	H	OH	H	H	OH	H
Apigenin 7-O-β-D-glucoside (cosmoside) (**2**)	H	H	O-glc	H	H	OH	H
Apigenin 7-O-[6′″-O-acetyl]-β-D-allosyl-(1→2)-β-D-glucoside (**3**)	H	H	O-[6′″-acetyl-allosyl]-(1→2)-glc	H	H	OH	H
Apigenin 7-(3″-E-*p*-coumaroyl)-β-D-glucoside (**4**)	H	H	O-(3″-E-*p*-coumaroyl)-glc	H	H	OH	H
Apigenin 7-(3″-Z-*p*-coumaroyl)-β-D-glucoside (**5**)	H	H	O-(3″-Z-*p*-coumaroyl)-glc	H	H	OH	H
Apigenin 7-(6″-E-*p*-coumaroyl)-β-D-glucoside (**6**)	H	H	O-(6″-E-*p*-coumaroyl)-glc	H	H	OH	H
Apigenin 7-(6″-Z-*p*-coumaroyl)-β-D-glucoside (**7**)	H	H	O-(6″-Z-*p*-coumaroyl)-glc	H	H	OH	H
Apigenin 7-(3″,6″-*p*-dicoumaroyl)- β-D-glucoside (Anisofolin A) (**8**)	H	H	O-(3″,6″-*p*-dicoumaroyl)-glc	H	H	OH	H
Apigenin 8-C-glucoside (**9**)	H	H	OH	C-glc	H	OH	H
Apigenin 6,8-di-C-glucoside (Vicenin-2) (**10**)	H	C-glc	OH	C-glc	H	OH	H
Isoscutellarein (**11**)	H	H	OH	OH	H	OH	H
Isoscutellarein 7-O-glucoside (**11a**)	H	H	O-glc	OH	H	OH	H
4′-Methyl-isoscutellarein (**12**)	H	H	OH	OH	H	OCH_3_	H
Isoscutellarein 7-O-allosyl-(1→2)-glucoside (**13**)	H	H	O-allosyl-(1→2)- glc	OH	H	OH	H
3′-Hydroxy-isoscutellarein-7-O-[6′″-O-acetyl]-β-D-glucoside (**14**)	H	H	O-[6′″-O-acetyl]- glc	OH	OH	OH	H
Isoscutellarein 7-O-[6′″-O-acetyl]-β-D-allosyl-(1→2)-β-D-glucoside (**15**)	H	H	O-[6′″-O-acetyl]-allosyl-(1→2)-glc	OH	H	OH	H
Isoscutellarein 7-O-β-D-allosyl-(1→2)-[6″-O-acetyl]-β-D-glucoside (**16**)	H	H	O-[6″-O-acetyl]-allosyl-(1→2)-glc	OH	H	OH	H
Isoscutellarein 7-O-[6′″-O-acetyl]-β-D-allosyl-(1→2)-[6″-O-acetyl]- β-D-glucoside (**17**)	H	H	O-[6′″-O-acetyl]-allosyl-(1→2)-[6″-O-acetyl]-glc	OH	H	OH	H
Isoscutellarein 8-O-(6″-trans-*p*-coumaroyl)-β-D-glucoside (**18**)	H	H	OH	O-(6”-trans-*p*-coumaroyl)-glc	H	OH	H
4′-Methyl-isoscutellarein 7-O-β-D-allosyl-(1→2)-β-D-glucoside (**19**)	H	H	O-allosyl-(1→2)-glc	OH	H	OCH_3_	H
4′-Methyl- isoscutellarein 7-O-β-D-allosyl-(1→2)-[6″-O-acetyl]-β-D-glucoside (**20**)	H	H	O-allosyl-(1→2)-[6″-O-acetyl]-glc	OH	H	OCH_3_	H
4′-Methyl-isoscutellarein 7-O-β-D-[6′″-O-acetyl]-allosyl-(1→2)-β-D-glucoside (**21**)	H	H	O-[6′″-O-acetyl]-allosyl-(1→2)-glc	OH	H	OCH_3_	H
4′-Methyl-isoscutellarein 7-O- [2″-O-acetyl]-β-D-allosyl-(1→2)-β-D-glucoside (**22**)	H	H	O-[2″-O-acetyl]-allosyl-(1→2)-glc	OH	H	OCH_3_	H
4′-Methyl-isoscutellarein 7-O-β-D-[4′′′-O-acetyl]-allosyl]-(1→2)-β-D-glucoside (annuoside) (**23**)	H	H	O-[4′′′-O-acetyl]-allosyl-(1→2)-glc	OH	H	OCH_3_	H
4′-Methyl-isoscutellarein 7-O-[6′′′-O-acetyl]-allosyl-(1→2)-[6″-O-acetyl]-glucoside (**24**)	H	H	O-[6′′′-O-acetyl]-allosyl-(1→2)-[6″-O-acetyl]-glc	OH	H	OCH_3_	H
Isostachyflaside (**25**)	H	H	OH	OH	H	O-mannosyl- (1→2)-glc	H
Acetyl-isostachyflaside (**26**)	H	H	OH	OH	H	O-[acetyl]-mannosyl- (1→2)-glc	H
Di-acetyl- isostachyflaside (**27**)	H	H	OH	OH	H	O-[diacetyl-mannosyl]- (1→2)-glc	H
Spectabiflaside (**28**)	H	H	O-mannosyl- (1→2)-glc	OH	OCH_3_	OH	H
Scutellarein (**29**)	H	OH	OH	H	H	OH	H
Scutellarein 7-O-β-D-glucoside[5,6, 4′-trihydroxyflavone-7-O-β-D-glucoside] (**30**)	H	OH	O-glc	H	H	OH	H
Scutellarein 7-O-β-D-mannnosyl- (1→2)-β-D-glucoside (stachyflaside) (**31**)	H	OH	O-mannosyl- (1→2)-glc	H	H	OH	H
7-O-β-D-glucopyranosyl-5,6-dihydroxy-4′-methoxyflavone (Stachannin A) (**32**)	H	OH	O-glc	H	H	OCH_3_	H
4′-Methoxy-scutellarein 7-[O-β-D-mannosyl-(1→2)-β-D-glucoside (Stachannoside B) (**33**)	H	OH	O-mannosyl- (1→2)-glc	H	H	OCH_3_	H
Luteolin (**34**)	H	H	OH	H	OH	OH	H
Luteolin 7-methyl ether (**35**)	H	H	OCH_3_	H	OH	OH	H
Luteolin 7-O-β-D-glucuronide (**36**)	H	H	O-glcA	H	OH	OH	H
Luteolin 7-O-β-D-glucoside (**37**)	H	H	O-glc	H	OH	OH	H
Luteolin 6-C-glucoside (isoorientin) (**38**)	H	-C-glc	OH	H	OH	OH	H
Luteolin 7-O-[6′′′-O-acetyl]-allosyl-(1→2)-glucoside (**39**)	H	H	O-[6′′′-O-acetyl]-allosyl-(1→2)-glc	H	OH	OH	H
6,8 Di-C-β-D-glucopyranosyl luteolin (Lucenin-2) (**40**)	H	C-glc	OH	C-glc	OH	OH	H
3′,4′-Dimethyl-luteolin-7-O-β-D-glucoside (**41**)	H	H	O-glc	H	OCH_3_	OCH_3_	H
Chrysoeriol (**42**)	H	H	OH	H	OCH_3_	OH	H
Chrysoeriol 7-O-β-D-glucoside (**43**)	H	H	O-glc	H	OCH_3_	OH	H
Chrysoeriol 7-O-[6′′′-O-acetyl]-β-D-allosyl-(1→2)-glucoside (Stachyspinoside) (**44**)	H	H	O-[6′′′-O-acetyl]- allosyl-(1→2)-glc	H	OCH_3_	OH	H
Chrysoeriol 7-O-[6″-O-acetyl]-β-D-allosyl-(1→2)-glucoside (Isostachyspinoside) (**45**)	H	H	O-[6″-O-acetyl]- allosyl-(1→2)-glc	H	OCH_3_	OH	H
Chrysoeriol 7-(3″-E-*p*-coumaroyl)-β-D-glucoside (**46**)	H	H	O-(3″-E-*p*-coumaroyl)-glc	H	OCH_3_	OH	H
Chrysoeriol 7-(6″-E-*p*-coumaroyl)-β-D-glucoside (**47**)	H	H	O-(6″-E-*p*-coumaroyl)-glc	H	OCH_3_	OH	H
Hypolaetin (**48**)	H	H	OH	OH	OH	OH	H
Hypolaetin-7-O-glucoside (**49**)	H	H	O-glc	OH	OH	OH	H
Hypolaetin-7-O-glucuronide (**49a**)	H	H	O-glcA	OH	OH	OH	H
Hypolaetin 7-O-allosyl-(1→2)-glucoside (**50**)	H	H	O-allosyl-(1→2)-glc	OH	OH	OH	H
Hypolaetin 7-O-[6′′′-O-acetyl]-β-D-allosyl-(1→2)-β-D-glucoside (**51**)	H	H	O-[6′′′-O-acetyl]- allosyl-(1→2)- glc	OH	OH	OH	H
Hypolaetin 7-O-[6″-O-acetyl]-allosyl-(1→2)glucoside (**52**)	H	H	O-[6″-O-acetyl]- allossyl-(1→2)- glc	OH	OH	OH	H
Hypolaetin 7-O-[6′′′-O-acetyl]-allosyl-(1→2)-[6″-O-acetyl]-glucoside (**53**)	H	H	O-[6′′′-O-acetyl]-allosyl-(1→2)-[6″-O-acetyl]- glc	OH	OH	OH	H
Hypolaetin 7-O-[6′′′-O-acetyl]-allosyl-(1→2)-[3″-O-acetyl]-glucoside (**54**)	H	H	O-[6′′′-O-acetyl]-allosyl-(1→2)-[3″-O-acetyl]- glc	OH	OH	OH	H
4′-Methyl-hypolaetin-7-O-allosyl-(1→2)-glucoside (**55**)	H	H	O-allosyl-(1→2)-glc	OH	OH	OCH_3_	H
4′-Methyl-hypolaetin-7-O-[6′′′-O-acetyl]-β-D-allopyranosyl-(1→2)-β-D-glucopyranoside (**56**)	H	H	O-[6′′′-O-acetyl]-allosyl-(1→2)- glc	OH	OH	OCH_3_	H
4′-Methyl-hypolaetin-7-O-[6″-O-acetyl]-β-D-allopyranosyl-(1→2)-β-D-glucopyranoside (**57**)	H	H	O-[6″-O-acetyl]-allosyl-(1→2)- glc	OH	OH	OCH_3_	H
4′-Methyl-hypolaetin-7-O-[6′′′-O-acetyl]-allosyl-(1→2)-[6″-O-acetyl]-glucoside (**58**)	H	H	O-[6′′′-O-acetyl]-allosyl-(1→2)-[6″-O-acetyl]- glc	OH	OH	OCH_3_	H
Selgin 7-O-glucoside (**59**)	H	H	O-glc	H	OCH_3_	OH	OH
Tricin 7-O-glucuronide (**60**)	H	H	O-glcA	H	OCH_3_	OH	OCH_3_
Tricin 7-O-glucoside (**61**)	H	H	O-glc	H	OCH_3_	OH	OCH_3_
Tricetin 3′,4′,5′-trimethyl-7-O-glucoside (**62**)	H	H	O-glc	H	OCH_3_	OCH_3_	OCH_3_
**R=** **O-glcA-glc (2** **→** **1)**							
Palustrin (**63**)	H	OH	OCH_3_	H	H	H	H
**R=** **O-glcA**							
Palustrinoside (**64**)	H	OH	OCH_3_	H	H	H	H

glc: glucose, glcA: glucuronide.

**Table 17:**
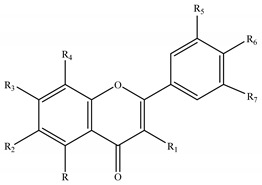
Chemical structures of poly-methylated flavonoids from *Stachys* spp.

Name	R_1_	R_2_	R_3_	R_4_	R_5_	R_6_	R_7_
R=OH							
Velutin (luteolin 7,3′-dimethyl ether) (**65**)	H	H	OCH_3_	H	OCH_3_	OH	H
Cirsimaritin (**66**)	H	OCH_3_	OCH_3_	H	H	OH	H
5,7,3′-Trihydroxy-6,4′-dimethoxyflavone (**67**)	H	OCH_3_	OH	H	OH	OCH_3_	H
5,7,3′-Trihydroxy-6,8,4′-trimethoxyflavone (**68**)	H	OCH_3_	OH	OCH_3_	OH	OCH_3_	H
Xanthomicrol (**69**)	H	OCH_3_	OCH_3_	OCH_3_	H	OH	H
Sideritiflavone (**70**)	H	OCH_3_	OCH_3_	OCH_3_	OH	OH	H
8-Methoxycirsilineol (**71**)	H	OCH_3_	OCH_3_	OCH_3_	OCH_3_	OH	H
Eupatorin (**72**)	H	OCH_3_	OCH_3_	H	OH	OCH_3_	H
Eupatilin (**72a**)	H	OCH_3_	OH	H	OCH_3_	OCH_3_	H
Eupatilin-7-methyl ether (**73**)	H	OCH_3_	OCH_3_	H	OCH_3_	OCH_3_	H
Salvigenin (**74**)	H	OCH_3_	OCH_3_	H	H	OCH_3_	H
5-Hydroxy-6,7,8,3′,4′-pentamethoxyflavone (**75**)	H	OCH_3_	OCH_3_	OCH_3_	OCH_3_	OCH_3_	H
5, 4′-Dihydroxy - 6,7,8,3′-tetramethoxyflavone (**76**)	H	OCH_3_	OCH_3_	OCH_3_	OCH_3_	OH	H
5, 4′-Dihydroxy-7,3′,5′-trimethoxyflavone (**77**)	H	H	OCH_3_	H	OCH_3_	OH	OCH_3_
Viscosine (5,7,4′-trihydroxy-3,6-dimethoxyflavone) (**78**)	OCH_3_	OCH_3_	OH	H	H	OH	H
Kumatakenin (kaempferol 3,7-dimethyl ether) (**79**)	OCH_3_	H	OCH_3_	H	H	OH	H
Pachypodol (quercetin 3,7,3′-trimethyl ether) (**80**)	OCH_3_	H	OCH_3_	H	OCH_3_	OH	H
Penduletin (**81**)	OCH_3_	OCH_3_	OCH_3_	H	H	OH	H
5,3′,4′-Trihydroxy-3,6,7,8-tetramethoxyflavone (**82**)	OCH_3_	OCH_3_	OCH_3_	OCH_3_	OH	OH	H
Calycopterin (**83**)	OCH_3_	OCH_3_	OCH_3_	OCH_3_	H	OH	H
Chrysosplenetin (**84**)	OCH_3_	OCH_3_	OCH_3_	H	OCH_3_	OH	H
5-Hydroxy-3,6,7,4′-tetramethoxyflavone (**85**)	OCH_3_	OCH_3_	OCH_3_	H	H	OCH_3_	H
5,8-Dihydroxy-3,6,7,4′-tetramethoxyflavone (**86**)	OCH_3_	OCH_3_	OCH_3_	OH	H	OCH_3_	H
Casticin (**87**)	OCH_3_	OCH_3_	OCH_3_	H	OH	OCH_3_	H
5-Hydroxy-3,6,7,8,4′- pentamethoxyflavone (5-hydroxyauranetin) (**88**)	OCH_3_	OCH_3_	OCH_3_	OCH_3_	H	OCH_3_	H
5,4′-Dihydroxy -3,6,7,8,3′- pentamethoxyflavone (**89**)	OCH_3_	OCH_3_	OCH_3_	OCH_3_	OCH_3_	OH	H
**R=OCH_3_**							
4′-Hydroxy- 3,5,7,3′-tetramethoxyflavone (**90**)	OCH_3_	H	OCH_3_	H	OCH_3_	OH	H

**Table 18:**
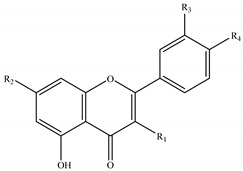
Chemical structures of flavonols from *Stachys* spp.

Name	R_1_	R_2_	R_3_	R_4_
Kaempferol (**91**)	OH	OH	H	OH
Isorhamnetin (**92**)	OH	OH	OCH_3_	OH
Quercetin 3-O-rutinoside (**93**)	O-rut	OH	OH	OH
Isorhamnetin 3-O-rutinoside (**94**)	O-rut	OH	OCH_3_	OH

rut: rutinoside.

**Table 19:**
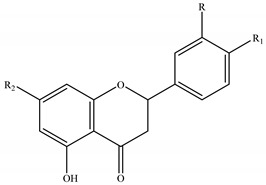
Chemical structures of flavanones from *Stachys* spp.

Name	R	R_1_	R_2_
Eriodictyol (**95**)	OH	OH	OH
Naringenin (**96**)	H	OH	OH
Hesperidin (**97**)	OH	OCH_3_	O-rut

rut: rutinoside.

**Table 20:** Chemical structure of biflavonoid from *Stachys* spp.

Stachysetin (98)
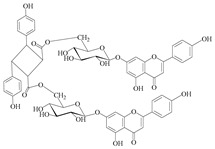

**Table 21:** Chemical structures of phenolic derivatives from *Stachys* spp.

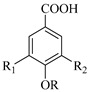		4-Hydroxybenzoic acid R=H, R_1_=H, R_2_=H (**99**)
		Vanillic acid R=H, R_1_=H, R_2_=OCH_3_ (**100**)
		Syringic acid R=H, R_1_= OCH_3_, R_2_=OCH_3_ (**101**)
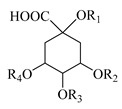		1-Caffeoylquinic acid R_1_=caffeoyl-, R_2_=R_3_=R_4_=H (**102**)
		3-Caffeoylquinic acid (Chlorogenic acid) R_1_=H, R_2_=caffeoyl-, R_3_=R_4_=H (**103**)
		4-Caffeoylquinic acid (cryptochlorogenic acid) R_1_=R_2_=H, R_3_=caffeoyl-, R_4_=H (**104**)
		5-Caffeoylquinic acid (neohlorogenic acid) R_1_=R_2_=R_3_=H, R_4_=caffeoyl- (**105**)
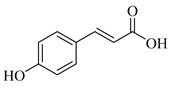 *p*-Coumaric acid (**106**)	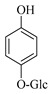 Arbutin (**107**)	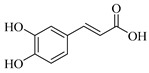 Caffeic acid (**108**)

Glc: glucose.

**Table 22:** Chemical structures of acetophenone glycosides from *Stachys* spp.

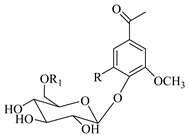
Androsin R=R_1_=H (**109**)
Neolloydosin R=H, R_1_=Xyl (**110**)
Glucoacetosyringone R=OCH_3,_ R_1_=H (**111**)

Xyl: xylose.

**Table 23:** Chemical structures of lignans from *Stachys* spp.

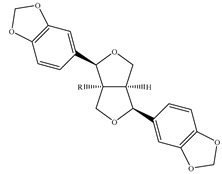	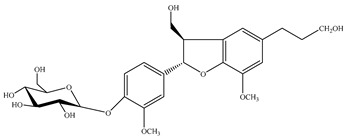
Sesamin R=H (**112**) Paulownin R=OH (**113**)	(7S-8R)-Urolignoside (**114**)

**Table 24:**
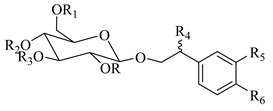
Chemical structures of phenylethanoid glycosides from *Stachys* spp.

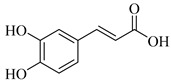 Caffeic acid			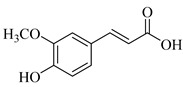 Ferulic acid				
**Name**	**R_1_**	**R_2_**	**R_3_**	**R_4_**	**R_5_**	**R_6_**	**R**
Rhodioloside (Salidroside) (**115**)	H	H	H	H	H	OH	H
Verbasoside (decaffeoyl-acteoside) (**116**)	H	H	Rha	H	OH	OH	H
2-Phenylethyl-D-xylopyranosyl-(1→6)-D-glucopyranoside (**117**)	Xyl	H	H	H	H	H	H
Acteoside (Verbascoside) (**118**)	H	Caf	Rha	H	OH	OH	H
Isoacteoside (**119**)	Caf	H	Rha	H	OH	OH	H
Darendoside B (deacyl-martynoside) (**120**)	H	H	Rha	H	OH	OCH_3_	H
β-OH-Acteoside (Campneoside II) (**121**)	H	Caf	Rha	OH	OH	OH	H
2′-O-Arabinosyl verbascoside (**122**)	H	Caf	Rha	H	OH	OH	Ara
Betonyoside A (**123**)	H	Fer	Rha	OH	OH	OH	H
Betonyoside B/C (isomers) (**124/125**)	Fer	H	Rha	OH	OH	OH	H
Betonyoside D (**126**)	Api	Cis-fer	Rha	H	OH	OCH_3_	H
Betonyoside E (**127**)	Api	Fer	Rha	OH	OH	OH	H
Betonyoside F (**128**)	H	Caf	Rha-Api	H	OH	OH	H
Lavandulifolioside A (Stachysoside A) (**129**)	H	Caf	Rha-Ara	H	OH	OH	H
Lavandulifolioside B (**130**)	H	4′-methyl-Fer	Rha-Ara	H	OCH_3_	OH	H
Leucosceptoside A (**131**)	H	Fer	Rha	H	OH	OH	H
Leucosceptoside B (**132**)	Api	Fer	Rha	H	OH	OCH_3_	H
Aeschynanthoside C (**133**)	H	Fer	Xyl	H	OH	OCH_3_	H
Leonoside B (Stachysoside D) (**134**)	H	Fer	Rha-Ara	H	OH	OCH_3_	H
Martynoside (**135**)	H	Fer	Rha	H	OH	OCH_3_	H
Campneoside I (**136**)	H	Caf	Rha	OCH_3_	OH	OH	H
Forsythoside B (**137**)	Api	Caf	Rha	H	OH	OH	H
β-OH-Forsythoside B methyl ether (**138**)	Api	Caf	Rha	OCH_3_	OH	OH	H
Leonoside A (Stachysoside B) (**139**)	H	Fer	Rha-Ara	H	OH	OH	H
* Stachysoside C (**140**)	H	Fer	Rha-Ara	H	OH	OH	H
Lamiophloside A (**141**)	Api	Fer	Rha	H	OCH_3_	OH	H
Parvifloroside A (**142**)	H	Caf	H	H	OH	OH	Rha
Parvifloroside B (**143**)	Caf	H	H	H	OH	OH	Rha

Caf: Caffeic acid, Fer: Ferulic acid, Api: Apioside, Rha: Rhamnoside, Ara: Arabinoside, Xyl: Xyloside, *: might be synonym of Leonoside B.

**Table 25:** Chemical structures of phenylpropanoid glucosides from *Stachys* spp.

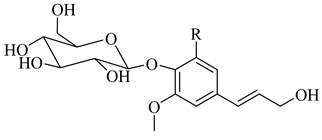	
Coniferin R=H (**144**)	Syringin R=OCH_3_ (**145**)

**Table 26:**
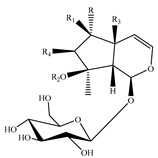
Chemical structures of iridoids from *Stachys* spp.

Name	R	R_1_	R_2_				R_3_	R_4_
Ajugol (**146**)	H	OH	H				H	H
Ajugoside (**147**)	H	OH	Ac				H	H
Harpagide (**148**)	H	OH	H				OH	H
7-Hydroxyharpagide (**149**)	H	OH	H				OH	OH
8-Acetylharpagide (Acetylharpagide) (**150**)	H	OH	Ac				OH	H
5-Desoxyharpagide (**151**)	OH	OH	H				H	H
5-Desoxy-8-acetylharpagide (**152**)	OH	OH	Ac				H	H
Reptoside (**153**)	H	H	Ac				OH	H
Harpagoside (**154**)	H	OH	Cinnamoyl-				OH	H
6-O-Acetylmioporoside (**155**)	AcO	H	H				H	H
Macranthoside (**156**)	H	OH	3,4-dimethoxy cinnamoyl-				OH	H
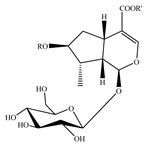	8-*Epi-*loganic acid R=R′=H (**157**)							
	7-O-Acetyl-8-*epi*-loganic acid R=Ac, R′=H (**158**)							
	8-*Epi*-loganin R=H, R′=CH_3_ (**159**)							
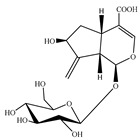	Gardoside (**160**)							
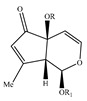	Allobetonicoside R=Allose, R_1_=Glc (**161**)							
	4′-O-β-D-galactopyranosyl-teuhircoside R=H, R_1_=Glc-Gal (**162**)							
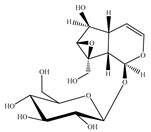	Catalpol (**163**)							
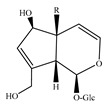	Aucubin R=H (**164**)							
	Monomelittoside R=OH (**165**)							
	Melittoside R=O-Glc (**166**)							
	5-O-Allopyranosyl-monomelittoside; 5-Allosyloxy-aucubin R=O-Alo (**167**)							
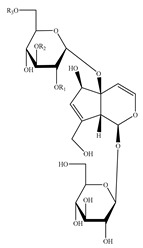	**Name**			**R_1_**	**R_2_**	**R_3_**		
	Stachysoside E (**168**)			H	*p*-(*E*)-coumaroyl-	H		
	Stachysoside F (**169**)			H	*p*-(*Z*)-coumaroyl-	H		
	Stachysoside G (**170**)			H	H	*p*-(*E*)-coumaroyl-		
	Stachysoside H (**171**)			*p*-(*E*)-coumaroyl-	H	H		
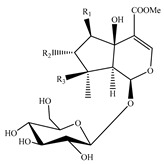	**Name**			**R_1_**	**R_2_**	**R_3_**		
	6β-Acetoxyipolamiide (**172**)			OAc	H	OH		
	6β-Hydroxyipolamiide (**173**)			OH	H	OH		
	Ipolamiide (**174**)			H	H	OH		
	Ipolamiidoside (**175**)			H	H	OAc		

Glc: Glucose, Gal: Galactose, Alo: Allose.

**Table 27:** Diterpenes from *Stachys* spp.

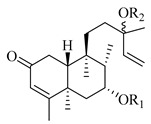					
Name		R_1_		R_2_	
Stachysolone (**177**)		H		H	
7-Monoacetyl-stachysolone (**178**)		Ac		H	
13-Monoacetyl-stachysolone (**179**)		H		Ac	
7,13-Diacetyl-stachysolone (**180**)		Ac		Ac	
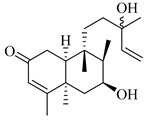		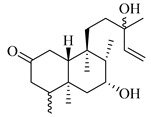		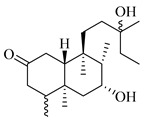	
Annuanone (**181**)		Stachylone (**182**)		Stachone (**183**)	
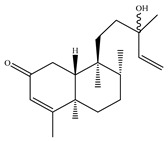		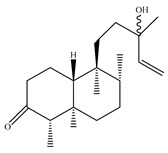		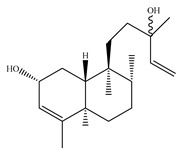	
Roseostachenone (**184**)		Roseostachone (**185**)		Roseostachenol (**186**)	
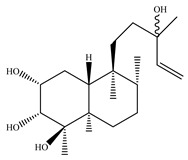				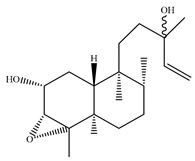	
Roseotetrol (**187**)				3α,4α-Epoxyroseostachenol (**188**)	
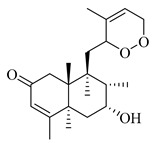					
Stachysperoxide (**189**)					
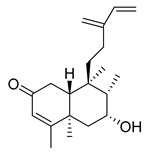				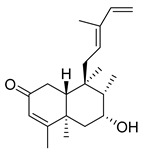	
Stachaegyptin A (**190**)				Stachaegyptin B (**191**)	
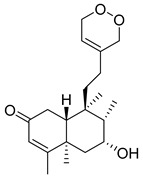				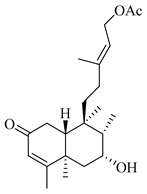	
Stachaegyptin C (**192**)				Stachaegyptin D (**193**)	
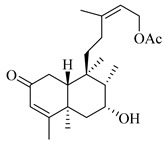				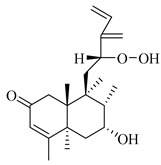	
Stachaegyptin E (**194**)				Stachaegyptin F (**195**)	
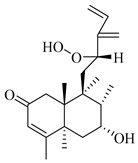				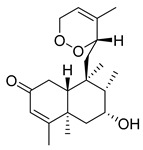	
Stachaegyptin G (**196**)				Stachaegyptin H (**197**)	
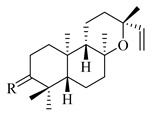				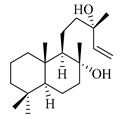	
Ribenone R=O (**198**) Ribenol R=αOH,βH(**199**)				13-*Epi*-sclareol (**200**)	
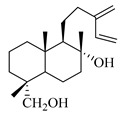				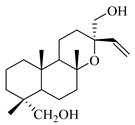	
(+)-6-Deoxyandalusol (**201**)				13-*Epi*-jabugodiol (**202**)	
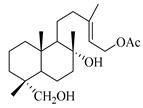					
(+)-Plumosol (**203**)					
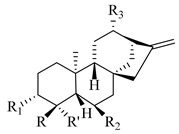					
**Name**	**R**	**R′**	**R_1_**	**R_2_**	**R_3_**
Stachysic acid (**204**)	COOH	CH_3_	H	OAc	H
6β-hydroxy-*ent*-kaur-16-ene (**205**)	CH_3_	CH_3_	H	OH	H
6β,18-dihydroxy-*ent*-kaur-16-ene (**206**)	CH_2_OH	CH_3_	H	OH	H
*Ent*-3α-acetoxy-kaur-16-en-19-oic acid (**207**)	CH_3_	COOH	OAc	H	H
3α,19-Dihydroxy-*ent*-kaur-16-ene (**208**)	CH_3_	CH_2_OH	OH	H	H
*Ent*-3α-hydroxy-kaur-16-en-19-oic acid (**209**)	CH_3_	COOH	OH	H	H
11a,18-Dihydroxy-*ent*-kaur-16-ene (**210**)	CH_2_OH	CH_3_	H	H	OH
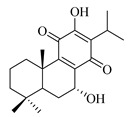 Horminone (**211**)					
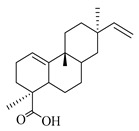 Stachyrosane 1 (**212**)			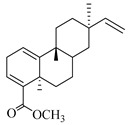 Stachyrosane 2 (**213**)		
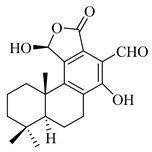 Betolide (**214**)			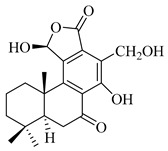 Betonicolide (**215**)		
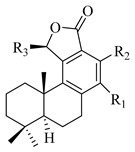		**Name**	**R_1_**	**R_2_**	**R_3_**
		Betonicoside A (**216**)	O-Glc	CH_2_OH	O-Glc
		Betonicoside B (**217**)	O-Glc	CH_2_OH	OH
		Betonicoside C (**218**)	OH	CH_2_OH	O-Glc
		Betonicoside D (**219**)	OH	CH_2_O-Glc	OH

Glc: Glucose.

**Table 28:** Triterpene derivatives, Phytosterols and Phytoecdysteroids from *Stachys* spp.

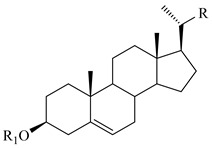	Stigmasterol (**220**)	R= 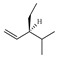	
			R_1_=H
	β-Sitosterol (**221**)	R= 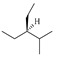	
			R_1_= H
	3-O-β-Sitosterol-glucoside (**222**)	R= 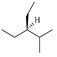	
			R_1_=Glc
** 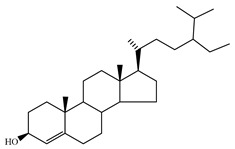 ** Lawsaritol (**223**)	** 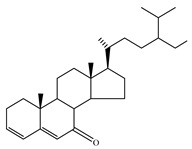 ** Stigmastan-3,5-dien-7-one (**224**)		
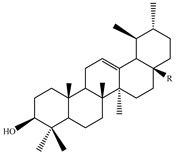 α-Amyrin R=CH_3_ (**225**) Ursolic acid R=COOH (**226**)	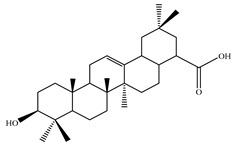 Oleanolic acid (**227**)		
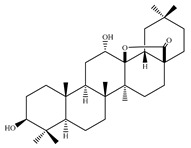 12α-hydroxy-oleanolic lactone (**228**)			
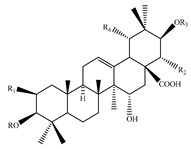			
Stachyssaponin A (**229**)	R=Glc-Rha, R_1_=H, R_2_=Glc-Ara, R_3_=H, R_4_=OH		
Stachyssaponin B (**230**)	R=Glc, R_1_=Ara, R_2_=H, R_3_=Glc, R_4_=H		
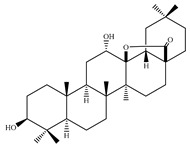	Stachyssaponin I R=OGlc-Ara, R_1_=Ara (**231**) Stachyssaponin II R=OGlc-Ara, R_1_=Ara-Rha (**232**) Stachyssaponin III R=OGlc-Xyl, R_1_=Ara-Rha (**233**) Stachyssaponin IV R=OGlc-Ara, R_1_=Ara-Rha-Xyl (**234**) Stachyssaponin V R=OGlc-Ara, R_1_=Ara-Rha-Xyl-^3^Ac (**235**) Stachyssaponin VI R=OGlc-Ara, R_1_= Ara-Rha-Xyl-^4^Ac (**236**) Stachyssaponin VII R=OGlc-Ara, R_1_=Ara-Rha-(^3^Glc)-Xyl (**237**) Stachyssaponin VIII R=OGlc-Xyl, R_1_=Ara-Rha-Xyl (**238**)		
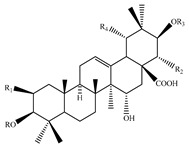	20-Hydroxyecdysone (**239**) R_1_=R_2_=R_3_=R_5_=H, R_4_=OH, R_6_=CH_3_		
	Polipodin B (**240**) R_1_=R_2_=R_5_=H, R_3_=R_4_=OH, R_6_=CH_3_		
	Integristeron A (**241**) R_2_=R_3_=R_5_=H, R_1_=R_4_=OH, R_6_=CH_3_		
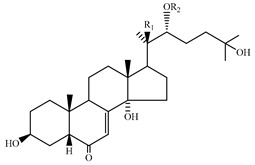	2-Desoxy-20-hydroxyecdysone (**242**) R_1_=OH, R_2_=H		
	2-Desoxyecdyson (**243**) R_1_=R_2_=H		

Glc: Glucose, Xyl: Xylose, Rha: Rhamnose, Ara: Arabinose.

**Table 29:** Chemical structures of megastigmane derivatives from *Stachys* spp.

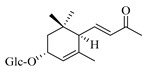	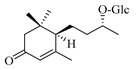
Byzantionoside A (**244**)	Byzantionoside B (**245**)
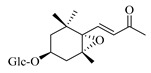	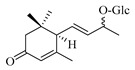
Icariside B_2_ (**246**)	(6R, 9R)- and (6R, 9S)-3-oxo-α-ionol glucosides (**247**)
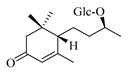	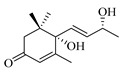
Blumeol C glucoside (**248**)	Vomifoliol (**249**)
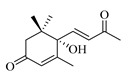	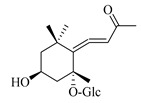
Dehydrovomifoliol (**250**)	Citroside A (**251**)

Glc: Glucose.

**Table 30:** Pharmacological activities of *Stachys* spp.

Species	Extract or Compound	Activity ^a^	Ref
*S. aegyptiaca* Pers.	Stachysolon diacetate (**180**)	**Cytotoxicity** HepG2 cell line IC_50_: 59.5 μM	[132]
*S. affinis* Bunge (=*S. sieboldii* Miq.)	Ethyl acetate fraction	**Antioxidant** DPPH IC_50_: 0.85 ± 0.04 μg/mL Superoxide radical scavenging activity: 38.63–61.41%	[28]
	Ethanol	**Cytotoxicity** K562 cell line; SH-SY5Y cell line; Caco-2 cell line: n.a. **Anti-ROS** K562 cell line; SH-SY5Y cell line; Caco-2 cell line EC_50_: 0.0023 mg/mL; 0.05 mg/mL; 0.026 mg/mL	[27]
*S. byzantina* K. Koch.	Methanol	**Antioxidant** Phosphomolybdenum (mmol TEs/g extract): 1.49 ± 0.12 ABTS (mg TEs/g extract): 143.85 ± 0.47 Nitric oxide (mmol TEs/g extract): 1.84 ± 0.02 CUPRAC (mg TEs/g extract): 134.73 ± 10.37	[153]
	Water	DPPH (mg TEs/g extract): 125.26 ± 1.47 Superoxide anion (mg TEs/g extract): 50.68 ± 2.05 FRAP (mg TEs/g extract): 98.73 ± 2.14 Chelating effect (mg EDTAEs/g extract): 16.69 ± 0.96	
	Ethyl acetate	**Anti-Alzheimer’s** AChE inhibition (mg GALAEs/g extract): 2.08 ± 0.01 BChE inhibition (mg GALAEs/g extract): 4.09 ± 0.04 **Anti-tyrosinase** Tyrosinase inhibition (mg KAEs/g extract): 33.27 ± 0.54 **Anti-diabetic** α-Amylase inhibition (mmol ACEs/g extract): 0.31 ± 0.01 α-Glucosidase inhibition (mmol ACEs/g extract): 1.95 ± 0.20	
*S. cretica* L. subsp. *smyrnaea* Rech. f.	Methanol	**Antioxidant** Ferrous ion chelating (mg EDTAEs/g dp): 4.82 ± 0.04 Phosphomolybdenum (mg TEs/g dp): 71.94 ± 4.56 DPPH (mg TEs/g dp): 9.10 ± 0.04 ABTS (mg TEs/g dp): 17.36 ± 0.07 CUPRAC (mg TEs/g dp): 14.67 ± 0.02 FRAP (mg TEs/g dp): 12.98 ± 0.11	[81]
	Methanol	**Anti-Alzheimer’s** AChE inhibition (µg GALAEs/g dp): 343.78 ± 10.79	
	Ethyl acetate	BChE inhibition (µg GALAEs/g dp): 167.68 ± 2.68	
	Ethyl acetate	**Anti-tyrosinase** Tyrosinase inhibition (mg KAEs/g dp): 2.45 ± 0.05	
	Methanol	**Anti-diabetic** α-Amylase inhibition (mg ACEs/g dp): 61.47 ± 0.05 α-Glucosidase inhibition (mg ACEs/g dp): 47.84 ± 0.78	
*S. cretica* L. subsp. *mersinaea* (Boiss.) Rech. f.	Water	**Antioxidant** Phosphomolybdenum (mmol TEs/g extract): 2.17 ± 0.21 DPPH (mg TEs/g extract): 176.21 ± 2.52	[108]
	Methanol	ABTS (mg TEs/g extract): 292.67 ± 1.53 CUPRAC (mg TEs/g extract): 256.79 ± 2.02 FRAP (mg TEs/g extract): 236.44 ± 2.96 Ferrous ion chelating (mg EDTAEs/g extract): 18.57 ± 0.04	
	Methanol	**Anti-Alzheimer’s** AChE inhibition (mg GALAEs/g extract): 2.03 ± 0.15	
	Ethyl acetate	BChE inhibition (mg GALAEs/g extract): 0.39 ± 0.01	
	Ethyl acetate	**Anti-tyrosinase** Tyrosinase inhibition (mg KAEs/g extract): 16.58 ± 0.31	
	Ethyl acetate	**Anti-diabetic** α-Amylase inhibition (mg ACEs/g extract): 396.50 ± 4.63	
	Methanol	α-Glucosidase inhibition (mg ACEs/g extract): 734.47 ± 4.32	
*S. cretica* L. subsp. *vacillans* Rech. f.	Methanol	**Antioxidant** (mg TE/g extract) DPPH: 191.47 ± 5.77 ABTS: 213.93 ± 21.83 CUPRAC: 579.23 ± 13.99 FRAP: 254.40 ± 8.58	[112]
	Water	Ferrous ion chelating (mg EDTAE/g extract): 68.72 ± 0.80	
	Methanol	**Anti-tyrosinase** Tyrosinase inhibition (mg KAE/g extract): 314.04 ± 2.05	
	Methanol	**Anti-diabetic** α-Amylase inhibition (mg ACE/g extract): 433.99 ± 5.10	
*S. ehrenbergii* Βoiss.	Methanol	**Antioxidant** ABTS IC_50_: 52 ± 7.5 mg/mL **Cytotoxicity** A549 cell line IC_50_: 420 ± 104 μg/mL	[154]
*S. glutinosa* L.	Dichloromethane; Xanthomicrol (**69**)	**Opioid Receptors binding affinity (*in silico*)** K_i_ for MOR = 10.3 μg/mL, K_i_ for DOR = 9.0 μg/mL; K_i_ for MOR = 0.83 μM, K_i_ for DOR = 3.6 μM **Antinociceptive (*in vivo*)**	[107]
*S. guyoniana* Noë ex Batt.	Chloroform *n-*Butanol Chloroform *n-*Butanol	**Antioxidant** β-carotene IC_50_: 2.30 ± 1.27 μg/mL DPPH IC_50_: 2.91 ± 0.14 μg/mL ABTS IC_50_: 7.29 ± 0.23 μg/mL CUPRAC A_0.50_: 0.15 ± 0.05 μg/mL Metal chelating assay (%) of inhibition at 100 μg/mL: 48.00 ± 1.71	[155]
	*n-*Butanol	**Anticholinesterase** AChE inhibition IC_50_: 5.78 ± 0.01 μg/mL BChE inhibition IC_50_: 39.10 ± 1.41 μg/mL	
	*n-*Butanol; Chloroform	**Antibacterial** MIC value: *S. aureus* 32 ± 0.90 μg/mL, *E. aerogenes* 32 ± 0.70 μg/mL; *E. coli* 64 ± 0.60 μg/mL	
*S. hissarica* Regel	-	**Wound Healing (** ***in vivo*** **)**	[67]
*S. iberica* var. *densipilosa* R. Bhattacharjee	Ethyl acetate;	**Antioxidant** ABTS (mg TEs/g extract): 138.16 ± 0.49, Nitric oxide (mmol TEs/g extract): 1.81 ± 0.01, Superoxide anion (mg TEs/g extract): 41.31 ± 1.64, CUPRAC (mg TEs/g extract): 111.47 ± 4.67;	[153]
	Water	DPPH (mg TEs/g extract): 82.52 ± 1.62 FRAP (mg TEs/g extract): 89.15 ± 0.82 Chelating effect (mg EDTAEs/g extract): 9.24 ± 0.87	
	Ethyl acetate	**Anti-Alzheimer’s** AChE inhibition (mg GALAEs/g extract): 2.16 ± 0.01 BChE inhibition (mg GALAEs/g extract): 4.20 ± 0.01 **Anti-tyrosinase** Tyrosinase inhibition (mg KAEs/g extract): 16.59 ± 0.33 **Anti-diabetic** α-Amylase inhibition (mmol ACEs/g extract): 0.34 ± 0.02 α-Glucosidase inhibition (mmol ACEs/g extract): 6.17 ± 0.51	
*S. iva* Griseb.	Stachysetin (**98**)	**Anti-diabetic (*in silico*)** Dipeptyl peptidase IV, peroxisome proliferator-active receptor gamma, aldose reductase, glycogen kinase, pancreatic alpha amylase precursor	[56]
*S. mialhesii* Noé	*n*-Butanol; Isoscutellarein-7-O-[6”′-O-acetyl]-β-D-allopyranosyl-(1→2)-β-D-glucoside (**15**)	**Antioxidant** DPPH IC_50_: 0.047 ± 0.0048 mg/mL; 0.066 ± 0.002 mg/mL	[103]
	*n*-Butanol	**Acute toxicity (*in vivo*)** Not toxic (10 g/kg of extract) **Antinociceptive(*in vivo*)** Inhibition of the writhing response induced by acetic acid (dose: 10,000; 5000 mg/kg) 77.11%, 58.22% **Antiinflammatory (*in vivo*)** Carrageenan-induced paw edema (dose: 5000 mg/kg) 52.03% **Ulcerogenic (*in vivo*)** n.a.	
*S. mucronata* Sieb.	*n*-Butanol fraction	**Anti-radical**	[156]
*S. lavandulifolia* Vahl.	Methanol Soxhlet extract; Arbutin (**107**), Ethanol; Arbutin (**107**), Methanol Soxhlet extract; Arbutin (**107**),	**Antioxidant** DPPH IC_50_: 25.0 ± 1.1 μg/mL; 62.5 ± 0.9 μg/mL, ABTS IC_50_: 19.9 μg/mL; 45.7 μg/mL, FRAP (μM Fe(II)/g): 44.5 ± 1.0; 12.2 ± 0.6,	[116]
	Methanol; Ethanol	β-carotene IC_50_: 29.3 µg/mL (30 min), 60.3 µg/mL (60 min); 33.0 µg/mL (30 min), 34.6 µg/mL (60 min)	
	Ethanol	**Anti-tyrosinase** Tyrosinase inhibition IC_50_: 33.4 ± 0.8 μg/mL	
	Hexane Dichloromethane	**Anti-Alzheimer’s** AChE inhibition IC_50_:13.7 ± 1.2 μg/mL BChE inhibition IC_50_: 143.9 μg/mL	
	Chloroform	**Cytotoxicity** Brine Shrimp lethality test: 121.8 ± 5.6 μg/mL	[13]
	Apigenin (**1**); Chrysosplenetin (**84**)	MRC-5 cell line IC_50_: 35.67 μg/mL; MDA-MB-231 cell line IC_50_: 88.23 μg/mL, HT-29 cell line IC_50_: 116.50 μg/mL	
*S. officinalis* (L.) Trevis (=*Betonica officinalis* L.)	Acetone Methanol	**Genotoxicity**	[157]
*S. ocymastrum* (L.) Briq. (=*S. hirta* L.)	6β-Acetoxyipolamiide (**172**); 6β-Hydroxyipolamiide (**173**); Ipolamiide (**174**); Ipolamiidoside (**175**)	**Antiangiogenic** **(*in vivo*)**	[123]
*S. parviflora * Benth. (=*Phlomidoschema parviflorum* (Benth.) Vved.)	Methanol	**Antioxidant** DDPH IC_50_: 76.87 ± 0.57 µg/mL BCB IC_50_: 188.47 ± 0.76 µg/mL **Cytotoxicity** A2780 cell line IC_50_: n.a HCT cell line IC_50_: n.a B16F10 cell line IC_50_: n.a **Antibacterial** MIC: *Bacillus cereus* 0.12 mg/mL	[64]
*S. pilifera* Benth.	Terpenoid fraction	**Cytotoxicity** HT29 cell line IC_50_: 46.44 μg/mL	[45]
	70% Methanol Alkaloid fraction	**Antiproliferative** Caspase-8 increased 99% Caspase-9 increased 85.38%	
	70% Ethanol	**Hepatoprotective (** ***in vivo*** **)**	[158]
	Hydroalcoholic	**Antioxidant** **(*in vivo*)** **Hepatoprotective** ***(in vivo)***	[159]
	Hydroalcoholic	**Antioxidant (*in vivo*)** **Renoprotective (*in vivo*)**	[19]
	Water	**Neuroprotective (*in vivo*)**	[152]
*S. riederi* var. *japonica* (Miq.) H. Hara	80% Ethanol	**Antioxidant/Cytoprotective** UVA-irradiated human dermal fibroblasts (HDFs) **Cytotoxicity** HDFs: l.a./n.a	[160]
*S. sieboldii* Miq. (=*S. affinis* Bunge)	*n*-Hexane fraction *n*-Hexane; 85% MeOH; *n*-BuOH; water fractions	**Antioxidant** ROS inhibition: 63% Increased GSH levels Inhbited oxidative DNA damage >90%	[29]
	(Root powder)	**Anti-obesity (*in vivo*)** **Anti-dyslipidemic *(in vivo)***	[161]
	20% Ethanol	**Memory protective (*in vivo*)**	[162]
*S. sylvatica* L.	Hydroalcoholic	**Polycystic ovary syndrome(*in vivo*)** (500 mg/kg) (mIU/mL), FSH 5.95 ± 0.02 mIU/mL, LH 6.48 ± 0.09 mIU/mL, Estrogen 0.9 ± 0.07 mIU/mL, LH/FSH 6.48/5.59 mIU/mL	[47]
*S. thirkei* K. Koch.	Methanol	**Antioxidant** β-carotene IC_50_: 47.79 ± 0.59 μg/mL DPPH IC_50_: 49.31 ± 0.38 μg/mL ABTS IC_50_: 13.34 ± 0.02 μg/mL CUPRAC absorbance%: 1.88 ± 0.02	[84]
	Acetone	**Anticholinesterase** AChE inhibition IC_50_: 52.46± 1.26% BChE inhibition IC_50_: 75.04 ± 1.91%	
	Methanol	**Cytotoxicity** A549 and L929 Fibroblast cells (100 mg/mL): n.a.	
	Acetone; Methanol	**Antimicrobial** Inhibition zone diameter: *S. aureus* (11 mm), *S. pyogenes* (10 mm), *E. coli* (10 mm), *P. aeruginosa* (n.a.), *C. albicans*: n.a.; *S. aureus* (10 mm), *S. pyogenes* (10 mm), *E. coli* (10 mm), *P. aeruginosa* (n.a.), *C. albicans*: n.a. MIC values: 250 ± 0.6 μg/mL, 300 ± 0.4 μg/mL, 250 ± 0.3 μg/mL, n.a., n.a.; 300 ± 0.1 μg/mL, 250 ± 0.2 μg/mL, 250 ± 0.4 μg/mL, n.a., n.a.	
*S. tmolea* Boiss.	Water	**Antioxidant** DPPH (mg TEs/g dp): 50.88 ± 1.55 ABTS (mg TEs/g dp): 44.39 ± 3.24 CUPRAC (mg TEs/g dp): 87.57 ± 0.83 FRAP (mg TEs/g dp): 51.80 ± 2.17 Phosphomolybdenum (mg TEs/g dp): 40.58 ± 3.45 Ferrous ion chelating (mg EDTAEs/g dp): 1.10 ± 0.03	[85]

^a^ Only the highest activity; n.a.:no activity; l.a.: low activity.

**Table 31:** General characteristics of the analyzed studies in the current review.

Type of Data	No of Studies *	Years of Publication
Ethnobotanical	48	since 1914
Phytochemical	91	since 1968
Pharmacological	22 (in vitro)	since 2015
	8 (in vivo)	
	2 (in silico)	
Clinical studies	4	since 2013
Reviews	4	since 1994

* N.B. It could be found more than one type of data in the same article.
